# Vertical targeting of the PI3K/AKT pathway at multiple points is synergistic and effective for non-Hodgkin lymphoma

**DOI:** 10.1186/s40164-024-00568-6

**Published:** 2024-11-01

**Authors:** Kristyna Kupcova, Jana Senavova, Filip Jura, Vaclav Herman, Anezka Rajmonova, Mariana Pacheco-Blanco, Tereza Chrbolkova, Iva Hamova, R. Eric Davis, Ondrej Havranek

**Affiliations:** 1 First Faculty of Medicine, BIOCEV, Charles University, Prumyslova 595, Prague, 25250 Czech Republic; 2 First Department of Medicine, Department of Hematology, First Faculty of Medicine, Charles University and General University Hospital, Prague, Czech Republic; 3 Department of Lymphoma and Myeloma, The University of Texas MD Anderson Cancer Center, Houston, TX USA

**Keywords:** Non-Hodgkin lymphoma, NHL, Diffuse large B-cell lymphoma, DLBCL, PI3K/AKT pathway, Inhibitors, Idelalisib, Ipatasertib, Rapamycin, PDPK1

## Abstract

The phosphatidylinositol 3‑kinase/protein kinase B (PI3K/AKT) signaling pathway is critically active in many cell types, both normal and neoplastic. Many small-molecule inhibitors targeting different levels of the PI3K/AKT pathway have been developed for cancer therapy, but their efficacy is reduced by compensatory pathway re-activation mechanisms, and their tolerability by toxic side effects. We studied this problem using cell lines representing diffuse large B-cell lymphoma (SUDHL-4 and OCI-Ly7), a genetically-encoded live-cell reporter of AKT activity, and 3 small-molecule inhibitors targeting different levels of the pathway: idelalisib (PI3Kδ), GSK2334470 (PDPK1), and ipatasertib (AKT). Half-maximal (IC50) concentrations of these inhibitors for AKT activity inhibition at 1 h, when used individually, were much lower than their IC50 values for reduction of viable cell number after 4 days. Time-course studies explained this discrepancy: AKT activity in the continuous presence of the inhibitors returned to normal after 24 h, and was supranormal after inhibitor removal. Combining all 3 inhibitors produced sustained inhibition of AKT activity, was broadly synergistic at reducing viable cell number, enabled substantially lower doses of each inhibitor to be used, and was enhanced further by the mTOR inhibitor rapamycin. Moreover, combined PDPK1 and AKT inhibition showed synergy with multiple different PI3K inhibitors. In a syngeneic mouse cell line model of lymphoma (A20), the triple combination showed antitumor activity and no evidence of toxicity. Our findings provide proof of concept suggesting further study of the safety and efficacy of low-dose multilevel PI3K/AKT pathway inhibition, for lymphoma and perhaps other cancers.

**Supplementary Information:**

The online version contains supplementary material available at 10.1186/s40164-024-00568-6.

**To the editor:** The phosphatidylinositol 3‑kinase/protein kinase B (PI3K/AKT) pathway promotes growth and survival in many cancers, and is important in many normal cells [1]. Small-molecule inhibitors of the PI3K/AKT pathway are approved for certain lymphoid neoplasms but are dose-limited by side effects, leading to low efficacy and/or resistance development [2–5].

## PI3K/AKT pathway inhibitors are more potent at reducing AKT activity than viable cell number

**Fig. 1:**
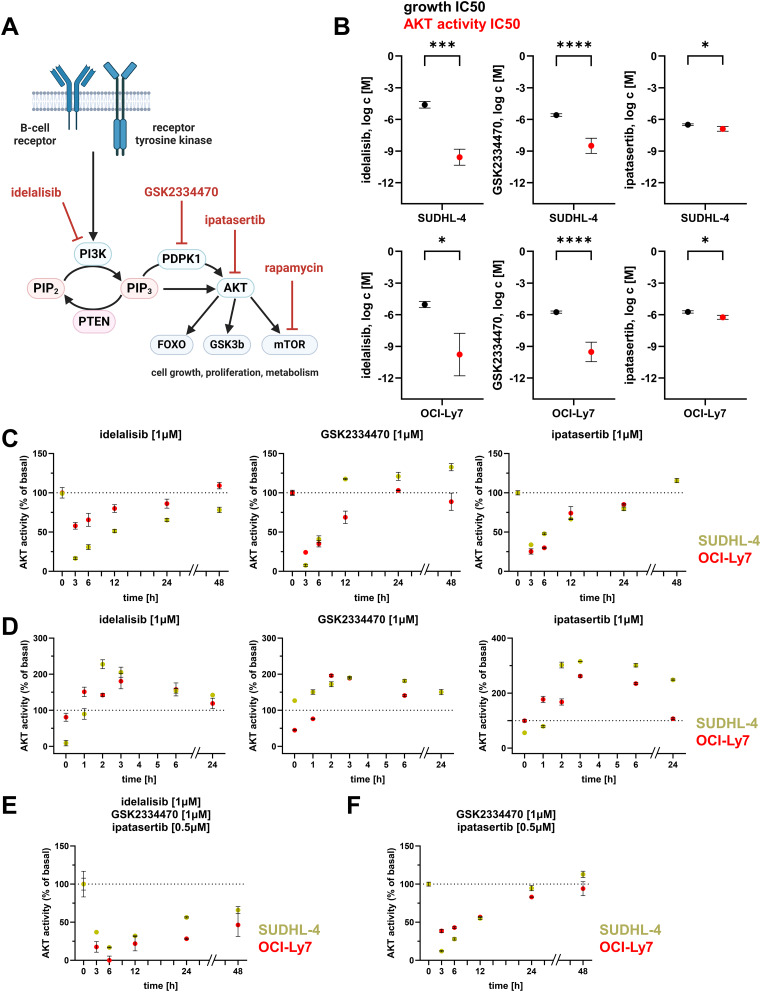
Dynamics of AKT activity after single or combined use of PI3K/AKT pathway-specific inhibitors in vitro. (**A**) Overview of the PI3K/AKT pathway highlighting inhibitors selected for combinatorial treatment. Created with BioRender.com. (**B**) Comparison between IC50 concentrations for AKT inhibition and cell growth reduction in DLBCL cell lines, based on data from Fig. S2 and S3. Three replicates with mean and standard deviation are displayed. (**C**) Long time course of AKT activity after exposure to individual inhibitors (at t = 0) showed near normalization of AKT activity by 24 h of in vitro treatment initiation. Three replicates with mean and standard deviation are displayed with significance determined by the unpaired *t*-test. (**D**) Strong counter-regulation of AKT activity was revealed by inhibitor release experiments. Following 24-hour pre-treatment with individual inhibitors, cells were washed and AKT activity showed a rapid and strong increase to supranormal levels. Three replicates with mean and standard deviation are displayed. (**E-F**) Long time course of AKT activity after exposure to inhibitor combinations (at t = 0) showed that combination of three inhibitors is necessary to achieve lasting AKT activity inhibition. In panel C-F, AKT activity was measured using FRET based AKT activity reporter and normalized to baseline, non-inhibited FRET efficiency (set as 100% AKT activity) and the FRET efficiency of non-responsive control reporter (0% AKT activity). Three replicates with mean and standard deviation are displayed. * *p* < 0.05, ** *p* < 0.01, *** *p* < 0.001, **** *p* < 0.0001

We hypothesized that PI3K/AKT pathway inhibition might be improved by combining inhibitors acting at different levels (Fig. 1A), which we term “vertical” combination. We first assessed PI3K/AKT pathway inhibitors for their individual ability to inhibit AKT activity measured with a Förster resonance energy transfer (FRET)-based biosensor [6, 7] in two cell lines representing diffuse large B-cell lymphoma. “Kinetic” monitoring showed that idelalisib (targeting the PI3Kδ isoform), GSK2334470 (PDPK1 inhibitor), and ipatasertib (pan-AKT inhibitor) decreased AKT activity within minutes (Fig. S1). However, for idelalisib and GSK2334470, the IC50 concentration for inhibition of cell growth after 4 days (Fig. S2) was much higher than the AKT-inhibitory IC50 concentration determined after 1 h incubations (Fig. S3 and 1B).

## Single PI3K/AKT inhibitors cause only transient decrease of AKT activity

We investigated these potency discrepancies of PI3K/AKT inhibitors by measuring AKT activity over longer times. Maximal inhibition was reached at 0.5–1 h, and sustained for ~ 6 h (Fig. S4). AKT activity rebounded thereafter, however, and reached essentially normal levels at 24 h (Fig. 1C). This increase was not due to inhibitor degradation: medium from treated cultures was equally able to inhibit AKT when cultured with untreated cells (Fig. S5).

Inhibitor-release experiments confirmed previous reports that compensatory responses to PI3K/AKT inhibitors normalize AKT activity [8]. Cells treated with individual inhibitors for 24 h, when washed and allowed to recover in inhibitor-free medium, showed a rapid increase in AKT activity to supranormal levels (Fig. 1D). Counter-regulation was also implied by the PI3K/AKT pathway inhibitor rapamycin, whose target (mTOR) is downstream of AKT; although rapamycin was effective at reducing cell growth (Fig. S2), it caused an increase in AKT activity (Fig. S3), as previously reported [9].

## Combined PI3K/AKT inhibitors provide lasting AKT activity inhibition and synergistic reduction in cell growth

**Fig. 2:**
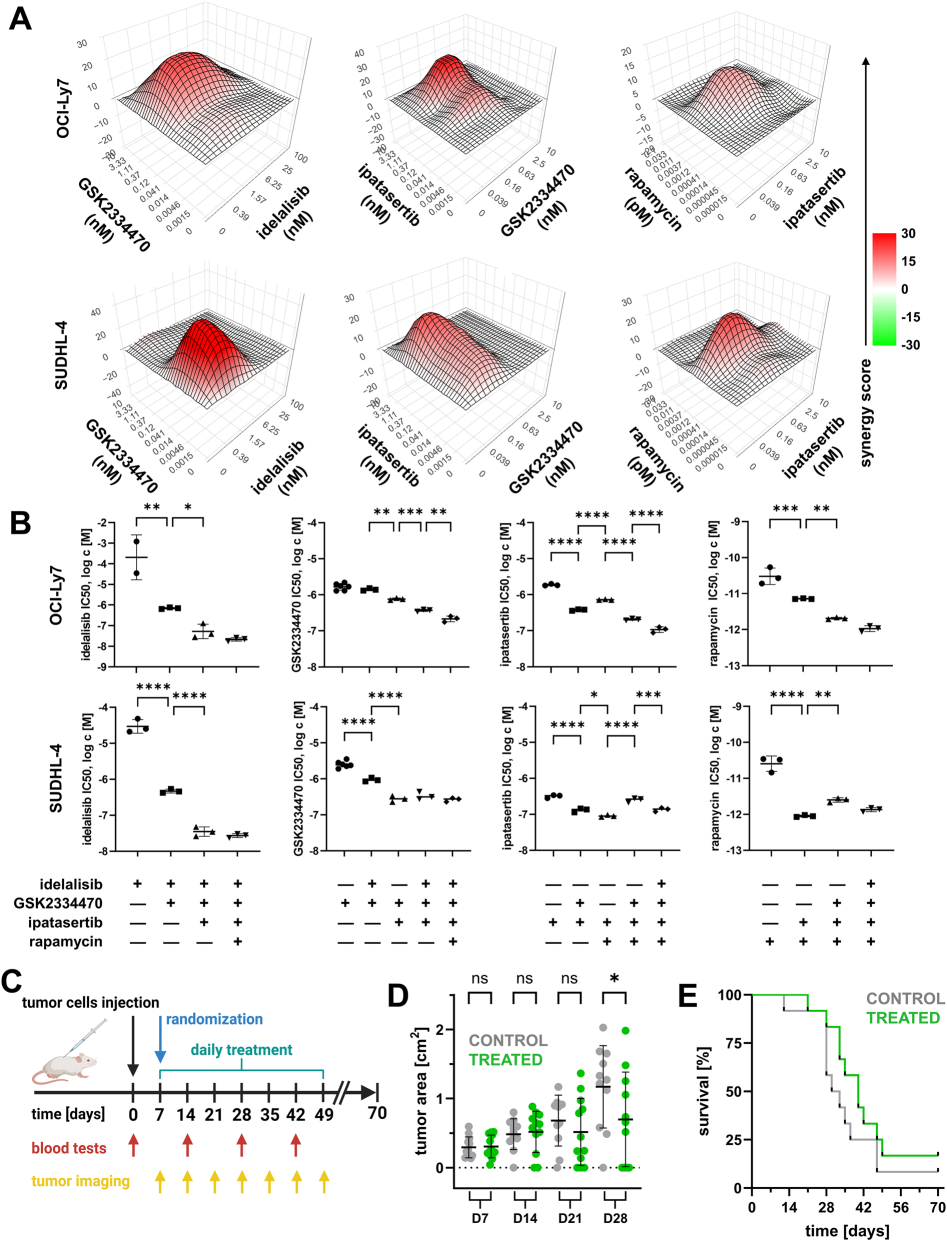
Vertical PI3K/AKT inhibition is synergistic, safe, and effective in vivo. (**A**) Drug synergy determination in pairwise combination of inhibitors GSK2334470, idelalisib, and ipatasertib after 96 h of incubation. Heatmap shows synergy score calculated using the zero interaction potency (ZIP) model (SynergyFinder3.0). Data are from one of three independent experiments (second and third replicates are displayed in Fig. S6). Regions of synergistic and antagonistic dose ratios are highlighted in red and green, respectively. Individual synergy scores are displayed in Tables S1-6 (**B**) Cell growth inhibitory IC50 concentrations for inhibitors compared to their cell growth inhibitory IC50 concentrations within inhibitor combinations. A fixed molar ratio of inhibitors was used, i.e., idelalisib : GSK : ipatasertib : rapamycin = 1 : 10 : 5 : 0.00005. Values are based on data displayed in Fig. S8. Three replicates with mean and standard deviation are displayed. Ordinary one-way ANOVA with multiple comparisons. (**C**) Schematics of in vivo experiment shows the timing of tumor cell injection, randomization, mice treatment, blood tests, and tumor size imaging. Created with BioRender.com. (**D**) Treatment reduced the tumor size at later timepoints. Mean and standard deviation are displayed. Ordinary one-way ANOVA with multiple comparisons. (**E**) Treatment also resulted in longer survival of treated mice in comparison to controls. Note: one treated mouse died without signs of tumor growth. * *p* < 0.05, ** *p* < 0.01, *** *p* < 0.001, **** *p* < 0.0001

GSK2334470 and ipatasertib produced lasting inhibition of AKT activity when combined with idelalisib (Fig. 1E), but not without idelalisib (Fig. 1F). When cross-titrations of pairs of PI3K/AKT inhibitors acting at different levels were evaluated for their effect on cell viability, all tested combinations showed strong synergy (Fig. 2A, Fig. S6, and Tables S1-18). In contrast, paired AKT inhibitors (ipatasertib and MK2206) showed little or no synergy (Fig. S7 and Tables S19-20).

## Vertical combination lowers the concentrations of PI3K/AKT pathway inhibitors needed for growth inhibition, and provides safe and effective antitumor activity in vivo

These results suggested that combining PI3K/AKT inhibitors would lower their growth-inhibitory IC50 concentrations. Through cross-titrations, we determined a highly-synergistic fixed molar ratio of inhibitors for further testing (see Supplement): idelalisib/GSK2334470/ipatasertib/rapamycin = 1/10/5/0.00005. When 2 to 4 inhibitors were used together in these ratios, IC50 concentrations were substantially decreased by each additional inhibitor (Fig. 2B and Fig. S8). Importantly, this synergism was not limited to a specific PI3K inhibitor; the GSK2334470/ipatasertib combination was similarly synergistic with idelalisib, duvelisib (PI3Kδ/γ inhibitor), copanlisib (pan-class I PI3K inhibitor), and umbralisib (next-generation PI3Kδ inhibitor; Fig. S9, Tables S21-28).

We tested vertical PI3K/AKT inhibition against syngeneic tumor growth in vivo (Fig. 2C), using the lymphoma cell line A20 in immune-competent BALB/c mice [10]. PI3K/AKT inhibitors were first tested for A20 growth inhibition in vitro; combination in the fixed molar ratio above reduced their IC50 concentrations, as compared to individual use (Fig. S10), from which in vivo dosing was determined (see Supplement). A20 was resistant to rapamycin, which was not used for in vivo experiments.

Tumor growth was significantly reduced by combination treatment (Fig. 2D, S11, and S12), resulting in longer survival (Fig. 2E). Vertical combination showed no side effects on body weight (Fig. S13), liver or renal function (Fig. S14), or blood counts (Fig. S15), including at the time of sacrifice (Fig. S16). This suggests that this triple combination is not toxic, and that higher doses might have been tolerated, perhaps improving tumor control.

## Discussion

Using a biosensor of AKT activity, we showed that initial efficacy of individual PI3K/AKT pathway inhibitors is lost within 24 h, due to previously-described compensatory activation [11]. Vertical combination of 3 or more inhibitors synergistically prevented this compensation, enabling sustained inhibition and toxicity to cancer cells at low concentrations. Clinically-approved inhibitors of multiple PI3K isoforms, and rapamycin as a 4th drug, were also shown to be synergistic in combinations. In vivo, combining two approved inhibitors (targeting PI3Kδ and AKT) with another in clinical trials (targeting PDPK1) showed anti-tumor efficacy without obvious toxicity, suggesting potential for clinical application. Furthermore, many cancers have distinctive upstream pathway activators that are targetable (Fig. 1A), such as kinases activated by the B-cell receptor in lymphoma, or receptor tyrosine kinases in carcinomas (e.g., EGFR or ERBB2).

Dose reduction by synergy may be especially important for PI3K inhibitors, “the ‘poster child’ for a drug class that should have been looking for a minimum-effective dose instead of a maximum-tolerated dose” [12]. Use of PI3K inhibitors has been limited by serious toxicities, likely due to their effect on PI3K (or another target) instead of AKT, since idelalisib used alone does not achieve sustained AKT inhibition. The synergy of vertical combinations may enable use of PI3K inhibitors at lower doses, reducing toxicity and increasing use of these potentially-valuable anticancer agents; however, more studies are needed, given the diverse spectrum of toxicities caused by PI3K inhibitors.

Previous studies of combined PI3K/AKT pathway inhibition in cancer have only targeted mTOR and another pathway member, with two inhibitors or a dual inhibitor [13–15]. Our results suggest that vertical combination of 3 or more agents may achieve the long-sought goal of tolerable but effective inhibition of the PI3K/AKT pathway.

## Electronic supplementary material

Below is the link to the electronic supplementary material.

Supplementary Material 1

Supplementary Material 2

## Data Availability

No datasets were generated or analysed during the current study.

## Abbreviations

AKT: Protein kinase B
DLBCL: Diffuse large B-cell lymphoma
FRET: Förster resonance energy transfer
NHL: non-Hodgkin lymphoma
PI3K: Phosphatidylinositol 3‑kinase
