# Supplementary material for: Vertical targeting of the PI3K/AKT pathway at multiple points is synergistic and effective for non-Hodgkin lymphoma
Source: Exp Hematol Oncol. 2024 Nov 1;13:108. doi: 10.1186/s40164-024-00568-6 (PMC11529427; doi:10.1186/s40164-024-00568-6)
Supplement: Supplementary file 1 — Supplementary Material 1 [file 40164_2024_568_MOESM1_ESM.pdf]

METHODS AND SUPPLEMENTARY FIGURES

Vertical Targeting of the PI3K/AKT Pathway at Multiple Points is Synergistic and Effective for Non- Hodgkin Lymphoma

Kristyna Kupcova[1,2], Jana Senavova[1,2], Filip Jura[1], Vaclav Herman[1,2], Anezka Rajmonova[1], Mariana Pacheco-Blanco[1], Tereza Chrbolkova[1], Iva Hamova[1,2], R. Eric Davis[3], and Ondrej Havranek[1,2]

[1] BIOCEV, First Faculty of Medicine, Charles University, Prague, Czech Republic

[2] First Department of Medicine – Department of Hematology, First Faculty of Medicine, Charles University and General University Hospital, Prague, Czech Republic

[3] Department of Lymphoma and Myeloma, The University of Texas MD Anderson Cancer Center, Houston, TX, U.S.A.

Correspondence: Ondrej Havranek, BIOCEV, First Faculty of Medicine, Charles University, Prumyslova 595, 25250, Czech Republic, +420 325873029, ondrej.havranek@lf1.cuni.cz

METHODS

Cell lines and culture conditions

For our study, we used 2 human DLBCL cell lines (OCI-Ly7 and SUDHL-4, *in vitro* experiments) and 1 mouse DLBCL cell line (A20) derived from BALB/cAnN (*in-vivo* experiments). All cell lines were cultured at 37 °C with 5% CO_2 in RPMI media with L-glutamine supplemented with: fetal bovine serum (FBS, final concentration 10%), HEPES buffer (final concentration 20 mM), sodium pyruvate (final concentration 1 mM), penicillin/streptomycin (final concentration 100 µg/mL) and 2-mercaptoethanol (final concentration 55 nM). All cell lines were validated by STR DNA fingerprinting (1). All cell lines were periodically tested for mycoplasma contamination using Mycoplasma PCR Detection Kit (Abm, Cat. No. G238).

Stable expression of AKT activity biosensor and mCardinal

We used the Sleeping Beauty transposon system (2) for stable expression of AKT activity biosensor and mCardinal fluorescent protein, similarly as we did before (1, 3). AKT activity biosensor plasmid (pSBbi-Pur-Lyn-AktAR2-EV, Addgene plasmid #125199) or its non-active variant plasmid (pSBbi-Pur-Lyn-AktAR2-EV-D, Addgene plasmid #125200) were co-electroporated together with a mammalian expression plasmid coding for SB100X transposase (pCMV(CAT)T7-SB100, Addgene plasmid #34879) (4), using 6 and 4 µg of plasmid DNA, respectively. For electroporation, we used the Neon electroporation device (Thermo Fisher Scientific). Electroporation and preparation of cell lines were done as we did previously (including parameters of electroporation) (1). For the 100 µL electroporation system, cells were prepared by changing the cell culture media every day for three days prior to the electroporation. On the day of electroporation cells were washed once with PBS, and 1.2 million cells were re-suspended in 120 µL of buffer R, mixed with midiprep plasmid DNA, and electroporated with the conditions optimal for each cell line (1). Cells were then re-suspended in 3 mL of pre-warmed antibiotic-free culture media. 24 hours after electroporation, puromycin was added into the media to a final concentration of 2 µg/mL and modified cells selected for 4 days. Similar approach was used to create mCardinal expressing A20 cell line. mCardinal cDNA was PCR amplified from pcDNA3-mCardinal (Addgene plasmid #51311) (5) and inserted into pSBbi-Pur (Addgene plasmid #60523) (2) using restriction based cloning.

Biosensor-based measurement of AKT activity

To precisely measure the AKT activity in live cells, we used a genetically encoded FRET-based AKT activity biosensor using flow cytometry-based FRET detection as we developed before (1, 3). The level of FRET is directly correlated to AKT activity. For all measurements, an unresponsive "dead" version of the reporter was used to control for artifactual non-specific changes in FRET and to set an AKT activity minimum. FRET detection for non-kinetic studies used a Cytoflex flow cytometer (Beckman Coulter), with default filters for individual FRET channels (405 nm laser with 450/50 filter for IDD and 525/50 filter for IDA; 488 nm laser with 530/30 filter for IAA). FRET kinetic studies were performed on a BD Fortessa flow cytometer (BD Biosciences), with custom filters for individual FRET channels (405 laser with 480/40 filter for IDD and 540/40 filter for IDA; 488 nm laser with 530/30 filter for IAA). Data were pre-processed in FlowJo software, exported as csv files and FRET efficiency calculated using moving median of FRET efficiency E over IAA in fRet R package as we described in detail previously (3).

Cell viability assays for drug sensitivity

Cell line suspensions at final concentration of $6x10^4$ cells/mL were plated in triplicate with drug dilution series in 96 well-plates in a final volume of 100 µL/well. After 96-hour incubation, cultures were analyzed to estimate the number of live cells using CellTiter-Glo assay (Promega, based on ATP detection) according to the manufacturer's instructions. Luminescence was measured on Tecan Infinite M200 PRO. Measured luminescence was corrected for background and data normalized for values in no-inhibitor control wells. Dose response curves were plotted and IC_{50} values calculated using non-linear regression (log(inhibitor) vs. response – variable slope (four parameters)) in GraphPad software. Inhibitors idelalisib, GSK2334470, ipatasertib and rapamycin were obtained from Selleck Chemicals.

Drug synergism testing and determination of optimal molar ratios

Detection of cell viability in cross titrations of 2 inhibitors was done similarly as for above-described single drug sensitivity testing. Two dilution series were cross set in 96 well plate. Cell suspensions of $6x10^4$ cells/mL were plated in triplicate with drug dilutions in 96 well-plates at 100 µL/well. After 96-hour incubation, cultures were analyzed to estimate the number of live cells using CellTiter-Glo assay (Promega, based on ATP detection) according to the manufacturer's instructions. Luminescence was measured on Tecan Infinite M200 PRO. Measured luminescence was corrected

for background and data normalized for values in no-inhibitor control wells. To calculate the drug synergistic effect, we used SynergyFinder online tool and zero interaction potency (ZIP) model (6, 7). Drug synergism testing allowed determination of the optimal molar ratio of each inhibitor pair. This was estimated from concentrations of inhibitors having the highest synergy. Setting optimal molar ratios was necessary for *in vitro* testing of more than two inhibitors and for the *in vivo* studies. Based on the optimal molar ratios of drug pairs, the combined four-drug molar ratio was determined as: idelalisib : GSK2334470 : ipatasertib : rapamycin = 1 : 10 : 5 : 0.00005.

In vivo experiments

For *in vivo* testing, we have used a syngeneic model of immune competent BALB/cAnNCrl mice and the lymphoma cell line A20 developed from this mouse strain (8). Prior to the *in vivo* testing, A20 cells were modified to stably express far red fluorescent protein mCardinal allowing *in vivo* imaging (5). On day 0 of the *in vivo* experiment (see the full schematics of *in vivo* experiment in Fig. 2C), 24 female mice (4-6 weeks old) were each subcutaneously injected into the left flank with $5x10^6$ A20 cells suspended in 200 µl of sterile PBS solution. Tumor growth was visualized every week with spectral imaging instrument Lago X Aura (Spectral Imaging Instrument, using same imager setup each time: exposure time: 2 s; binning: medium sets the pixel size of the CCD camera; Fstop: size 2 of the lens aperture; Xray: on; excitation: 605 nm; emission: 650 nm; excitation power 1). Tumor size was measured from acquired images Aura imaging software. Images were first all set to maximum radiance units: $2.21x10^7$; minimum radiance units: $6.15x10^6$; threshold radiance units: $6.15x10^6$ (radiance units – a calibrated absolute measurement of photon emission (photons/seconds/cm2/steradian) and area of any fluorescent signal counted as a tumor size. Moreover, tumor sizes were also measured three times per week using caliper in three diameters (tumor width, length, and depth) and tumor volume calculated as a product of 4 parameters: tumor width, length, depth, and the number π/6 (width x length x depth x (π/6)). All mice developed detectable tumor by day 7 and were randomly assigned into experimental and control groups (see comparison of tumor sizes between groups at day 7 in Fig. 2D).

Previously published single-agent dosing for individual inhibitors in mouse studies were 100 mg/kg/day orally for idelalisib (9), 40 mg/kg/day intra-peritoneally for GSK2334470 (10), and 40 mg/kg/day orally for ipatasertib (11). Dosing in humans for idelalisib (clinically approved 150 mg twice daily) and ipatasertib (200-400 mg daily in clinical trials) converts to 52 mg/kg/day in mice for idelalisib and to 35-70 mg/kg/day for ipatasertib in mice, respectively, according to guidelines for dose conversion between human and other animals (12). These converted doses are in a range

that is similar to those previously used in mouse models. Therefore, for our *in vivo* study, the previously-used *in vivo* dose for each inhibitor was adjusted using the ratio of *in vitro* IC50 for single inhibitor vs. IC50 of the inhibitor within combination (Fig. S10). Final dosing used for *in vivo* studies, therefore, were idelalisib 0.3 mg/kg daily orally, GSK2334470 6.1 mg/kg daily intra-peritoneally, and ipatasertib 2.3 mg/kg daily orally. These doses were, therefore, substantially lower than doses previously used for *in vivo* experiments: ~ 300-fold lower for idelalisib, ~ 7-fold lower for GSK2334470, and 18-fold lower for ipatasertib. The control group of mice received peroral and intraperitoneal administration of the same volume of vehicle only. For oral gavage, ipatasertib and idelalisib was dissolved in solution of sterile PBS with 40% of PEG300 and 1.22% of DMSO. For intraperitoneal application, GSK2334470 was dissolved in solution of sterile PBS with 40% of PEG300 and 1.32% of DMSO. Control group received daily oral and intraperitoneal applications of the same concentrations of PEG300 and DMSO only. Mice were sacrificed at the point where at least one of the three tumor dimensions exceeded 1.5 cm.

Blood samples were collected before experiment initiation and then every other week (see the experiment schematics in Fig. 2C) and at the time of mice termination. Beckman Coulter AU480 Clinical Chemistry Analyzer (Beckman Coulter, Inc.) was used to measure selected biochemical parameters (Total Bilirubin [µmol/L], Alanine aminotransferase [U/L], Aspartate aminotransferase [U/L], Urea [mmol/L], and Creatinine [µmol/L]) according to the manufacturer´s instructions. Mindray BC-5300 Vet Instrument (Mindray company) was used to measure basic hematology parameters including white blood cell count, platelet count, red blood cell count, hemoglobin level, lymphocyte count, and neutrophil count. Animal experiments were approved by the ethics committee of the Czech Academy of Science and performed exactly as within approved protocols. Tumor size growth was limited to 1.5 cm and all mice were sacrificed after any tumor diameter reached 1.5 cm. Mice experiments also adhered strictly to general guidelines regarding the use and welfare of laboratory animals.

Statistical analysis

Statistical analyses were performed using GraphPadPrism software (GraphPad Software Inc., San Diego, CA, USA). We used an unpaired *t*-test to compare two groups. For multiple-group comparisons, we used ordinary one-way ANOVA with Sidak's multiple comparisons test. For biochemical and hematological analysis results, we used Kruskal Wallis test with Dunn's multiple comparisons test. P value less than 0.05 was considered statistically significant. In figures: * $p < 0.05$, ** $p < 0.01$, *** $p < 0.001$, **** $p < 0.0001$.

SUPPLEMENTARY FIGURES

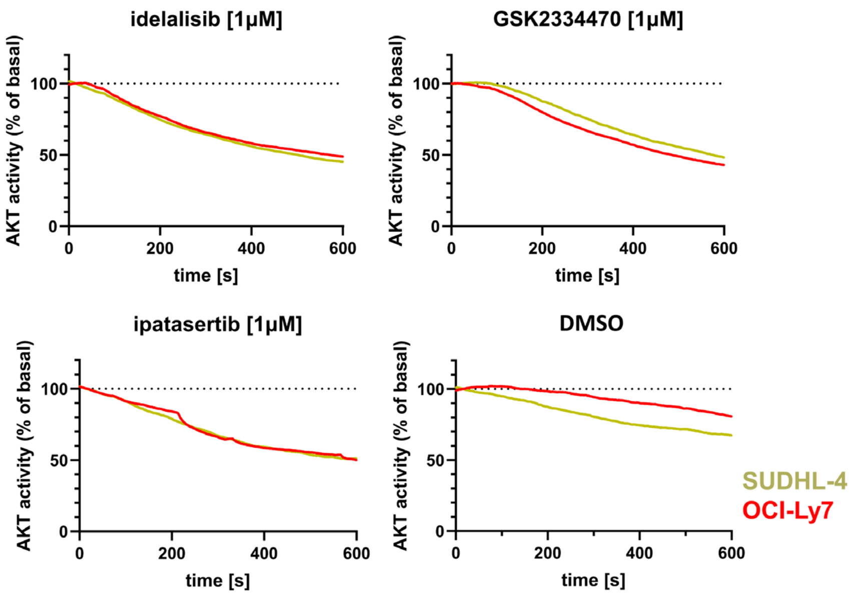

Figure S1. Kinetics of AKT inhibition for selected PI3K/AKT pathway inhibitors.

Kinetics of AKT activity shows its rapid decrease after addition of individual inhibitors. Inhibitors were added 30 seconds after measurement initiation. Vehicle only control (DMSO) showed smaller decrease of AKT activity. AKT activity was measured using FRET based AKT activity reporter and normalized to baseline, non-inhibited FRET efficiency (set as 100% AKT activity) and the FRET efficiency of non-responsive control reporter (0% AKT activity). All inhibitors (idelalisib, GSK2334470 or ipatasertib) were at 1µM final concentration. Two DLBCL cell lines (SUDHL-4 and OCI-Ly7) were used.

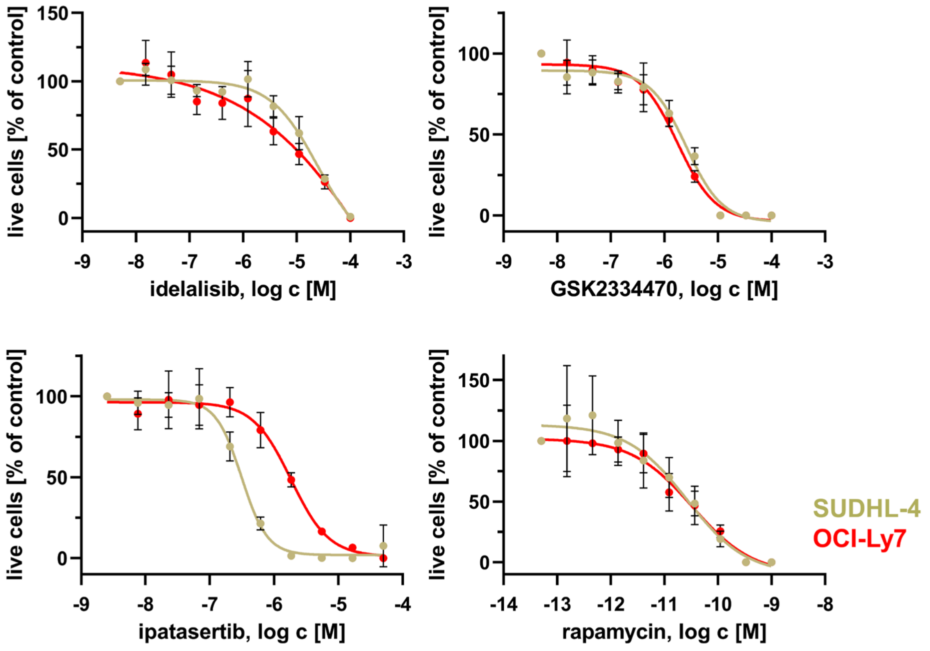

Figure S2. Determination of net growth inhibition IC50 concentration for selected PI3K/AKT pathway inhibitors.

Two DLBCL cell lines (SUDHL-4 and OCI-Ly7) were used. Number of live cells was normalized to no-inhibitor controls. Non-linear regression curve is displayed together with mean ± SD (n = 3). Original data for summary presentation in Fig. 1B.

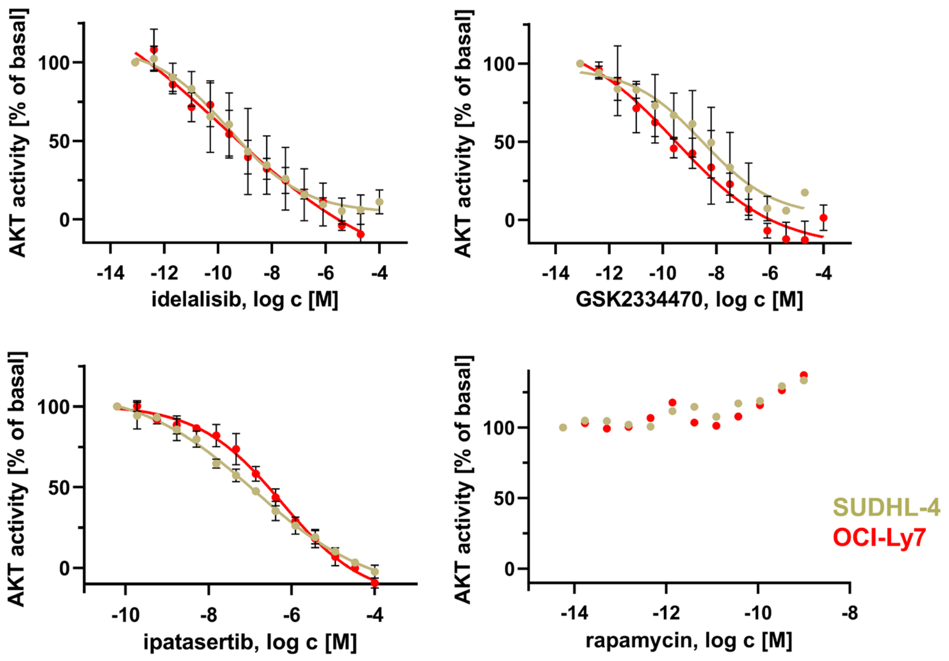

Figure S3. Determination AKT inhibitory effect of selected PI3K/AKT pathway inhibitors.

Dose-dependent reduction of AKT activity in two DLBCL cell lines (SUDHL-4 and OCI-Ly7) after 1-hour *in vitro* incubation with idelalisib, GSK2334470, ipatasertib, and rapamycin. Three replicates with means and SD are displayed, except for rapamycin (single replicate). AKT activity was measured using a FRET-based AKT activity reporter and normalized to the range between baseline, non-inhibited FRET efficiency (set as 100% AKT activity) and the FRET efficiency of a non-responsive control reporter (0% AKT activity). Original data for summary presentation in Fig. 1B

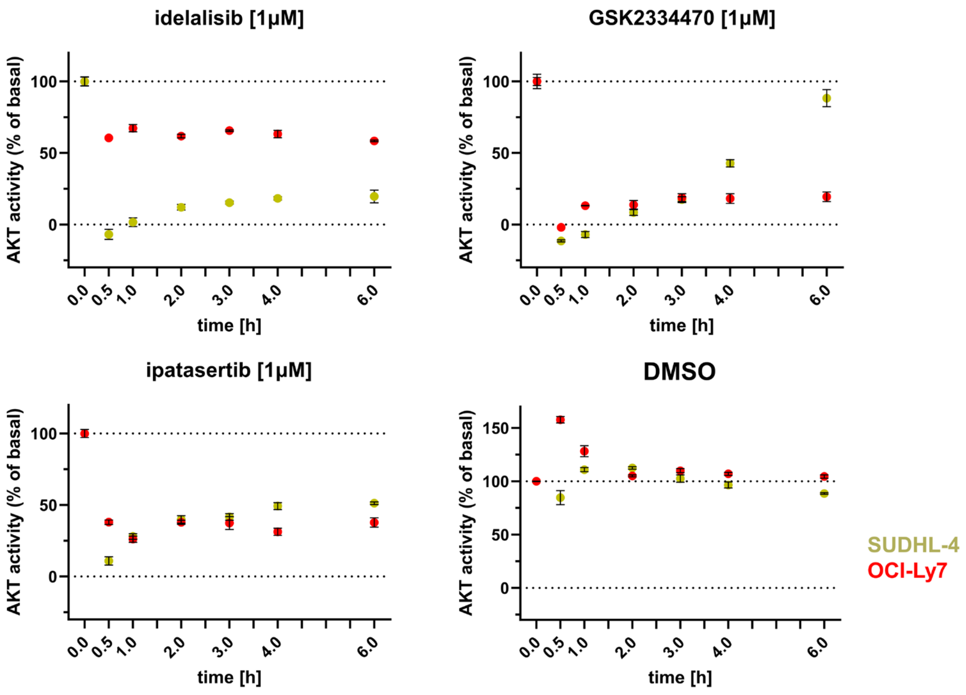

Figure S4. Short time course of AKT activity for selected PI3K/AKT pathway inhibitors

Short time course of AKT activity after exposure to individual inhibitors (at t = 0) showed their maximal AKT activity inhibitory effect at 30 minutes, followed by sustained AKT activity inhibition. AKT activity was measured using FRET based AKT activity reporter and normalized to baseline, non-inhibited FRET efficiency (set as 100% AKT activity) and the FRET efficiency of non-responsive control reporter (0% AKT activity). All inhibitors (idelalisib, GSK2334470 or ipatasertib) were at 1µM final concentration. Two DLBCL cell lines (SUDHL-4 and OCI-Ly7) were used. Three replicates with mean and standard deviation are displayed. For vehicle only control (DMSO), the same concentration of DMSO was used as with inhibitors.

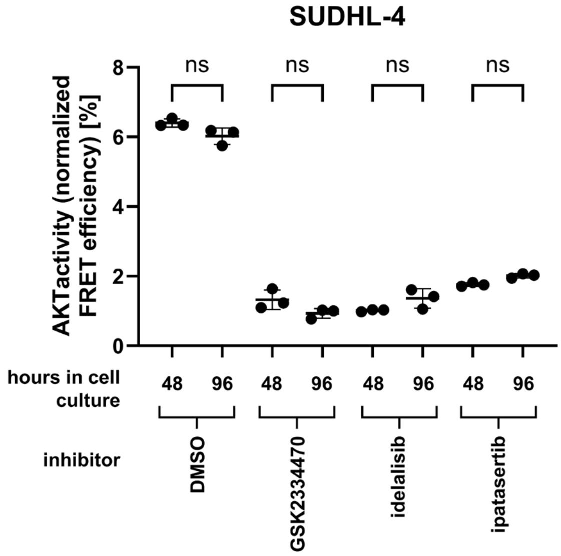

Figure S5. Selected PI3K/AKT pathway inhibitors are not degraded after 48 and 96 hours in cell culture.

SUDHL-4 cells were treated with selected PI3K/AKT inhibitors (all at 1µM concentration) or vehicle only (DMSO) for 48 and 96 hours and cell culture supernatants consequently used to treat fresh AKT activity reporter positive SUDHL-4 cells for 1 hour to determine the AKT inhibitory effect after inhibitors were for 48 and 96 hours in cell culture. AKT activity was measured using a FRET-based AKT activity reporter and normalized by subtraction of FRET efficiency of a non-responsive control reporter. Three replicates with means and SD are displayed. Ordinary one-way ANOVA with multiple comparisons.

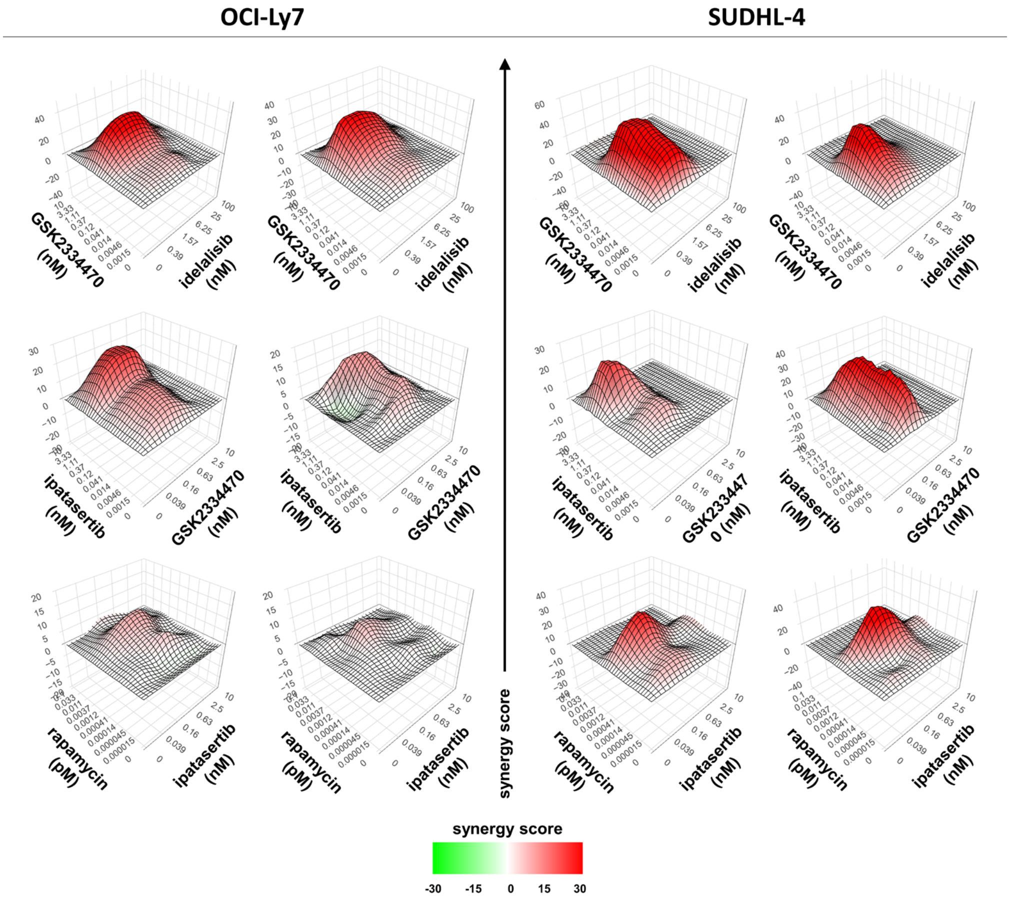

Figure S6. Drug synergy determination in pairwise combination of inhibitors.

Replicates of SUDHL-4 and OCI-Ly7 cell lines treated with GSK2334470, idelalisib and ipatasertib in combinations after 96 hours of incubation. Heatmap shows synergy calculation using zero interaction potency (ZIP) model synergy score (SynergyFinder3.0). Data are from 3 independent experiments (first replicate is displayed in Fig. 2A). Regions of synergetic and antagonistic dose ratios are highlighted in red and green, respectively. Individual synergy scores are displayed in Tables S7-18.

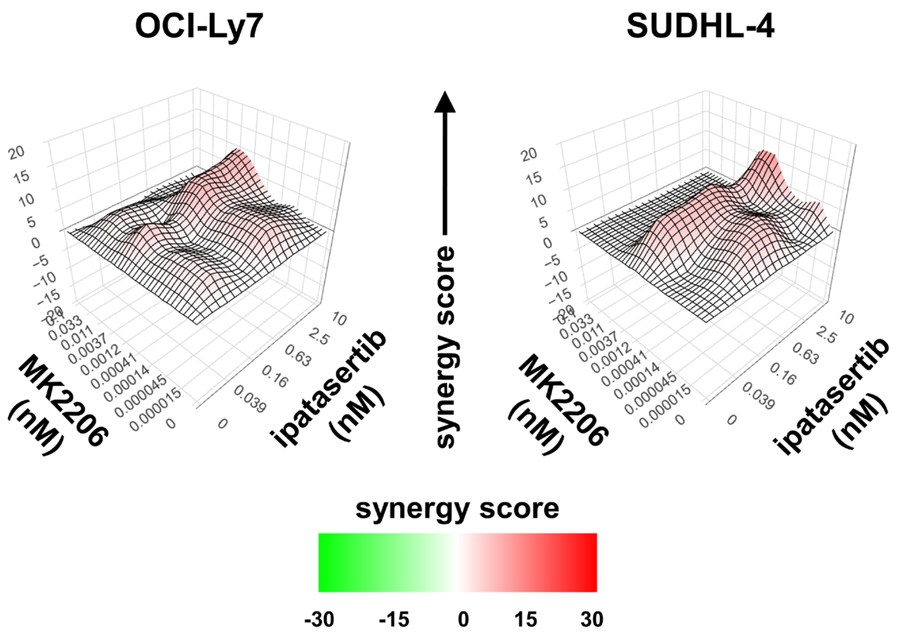

Figure S7. Synergy distribution in pairwise combination of two inhibitors of AKT.

Two DLBCL cell lines (SUDHL4 and OCI-Ly7) were treated with two different AKT inhibitors - MK2206 and ipatasertib in combinations for 96 hours. Heatmap depicts zero interaction potency (ZIP) model synergy score as calculated by SynergyFinder 3.0. Regions of synergetic and antagonistic dose ratios are highlighted in red and green, respectively. No significant synergy was found between two different inhibitors both targeting the same member of the PI3K/AKT pathway. Individual synergy scores are displayed in Tables S19-20.

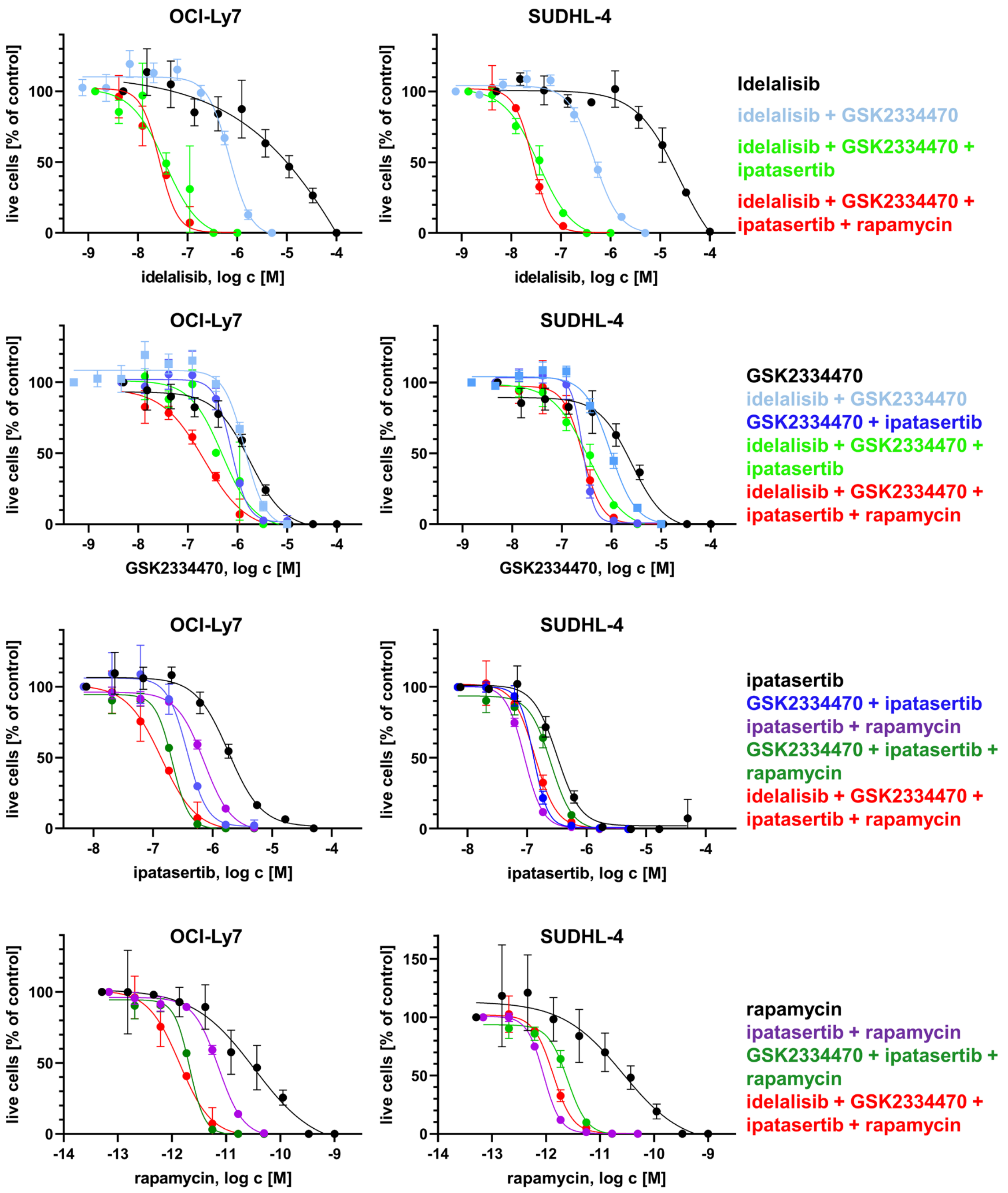

Figure S8. Determination of net growth inhibition IC50 concentrations for selected PI3K/AKT inhibitors and their combinations.

Dose response curves (n = 3 replicates) of single agent and their combinations were measured after 4 days inhibitor treatment of two DLBCL cell lines. For inhibitor combinations, fixed molar ratio of inhibitors was used - idelalisib : GSK : ipatasertib : rapamycin - 1 : 10 : 5 : 0.00005. Cell viability was measured using CellTiter-Glo assay and number of live cells was normalized to no-inhibitor controls. Non-linear regression curve is displayed together with mean ± SD (n = 3). Original data for summary presentation in Fig. 2B.

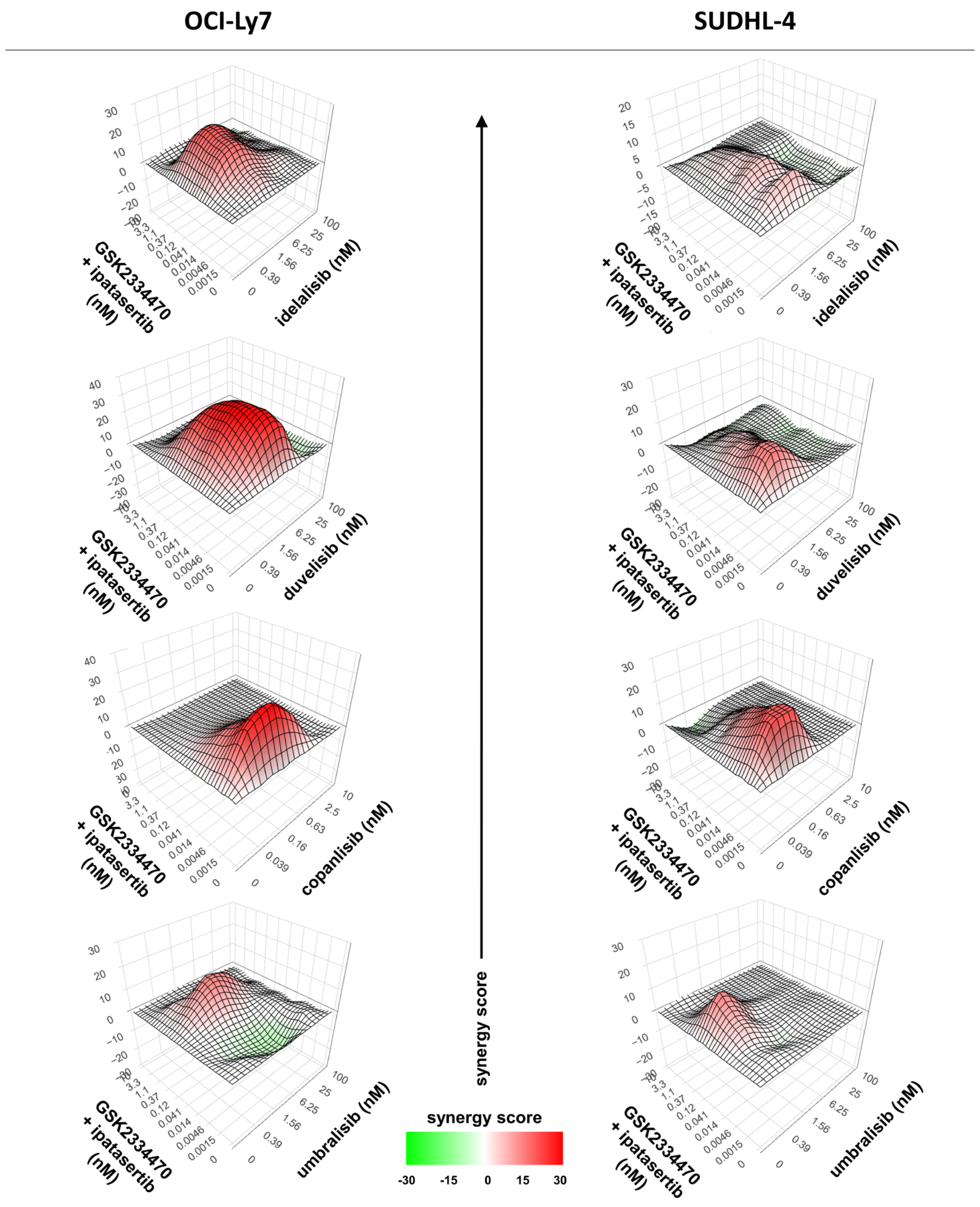

Figure S9. Drug synergy determination in pairwise combination of GSK2334470/ipatasertib mix and four different PI3K inhibitors.

SUDHL-4 and OCI-Ly7 cell lines were treated with GSK2334470/ipatasertib mix (in 2 : 1 molar ratio, respectively; concentrations are displayed for GSK2334470) and four different PI3K inhibitors: idelalisib (δ), duvelisib (δ/γ), copanlisib (pan), and umbralisib (next generation δ). Cell viability was measured after 96 hours of incubation. Heatmap shows synergy calculation using zero interaction potency (ZIP) model synergy score (SynergyFinder3.0). Individual synergy scores are displayed in Tables S21-28.

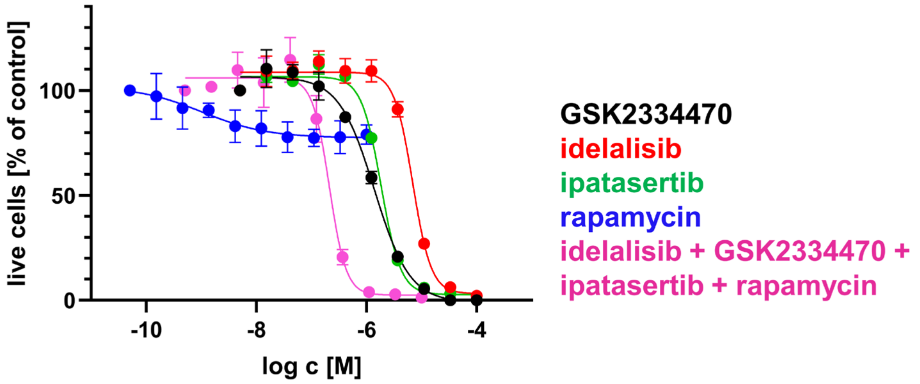

inhibitor	Log IC50 concentration for single inhibitors [M]	Log IC50 concentration of individual inhibitors in combination [M]	Ratio of concentrations (single inhibitor / in combination)
idelalisib	-5.16	-7.67	0.003
GSK2334470	-5.86	-6.67	0.152
ipatasertib	-5.73	-6.97	0.057

Figure S10. Determination of net growth inhibition IC50 concentration for selected PI3K/AKT pathway inhibitors and their combination in A20 cell line.

For inhibitors combination, fixed molar ratio of inhibitors was used - idelalisib : GSK2334470 : ipatasertib : rapamycin - 1 : 10 : 5 : 0.00005. Mouse lymphoma cell line A20. Number of live cells was normalized to no-inhibitor controls. Non-linear regression curve is displayed together with mean ± SD (n = 3). Table show IC50 concentrations for single inhibitors and individual inhibitors in their combination and ratio of these inhibitors.

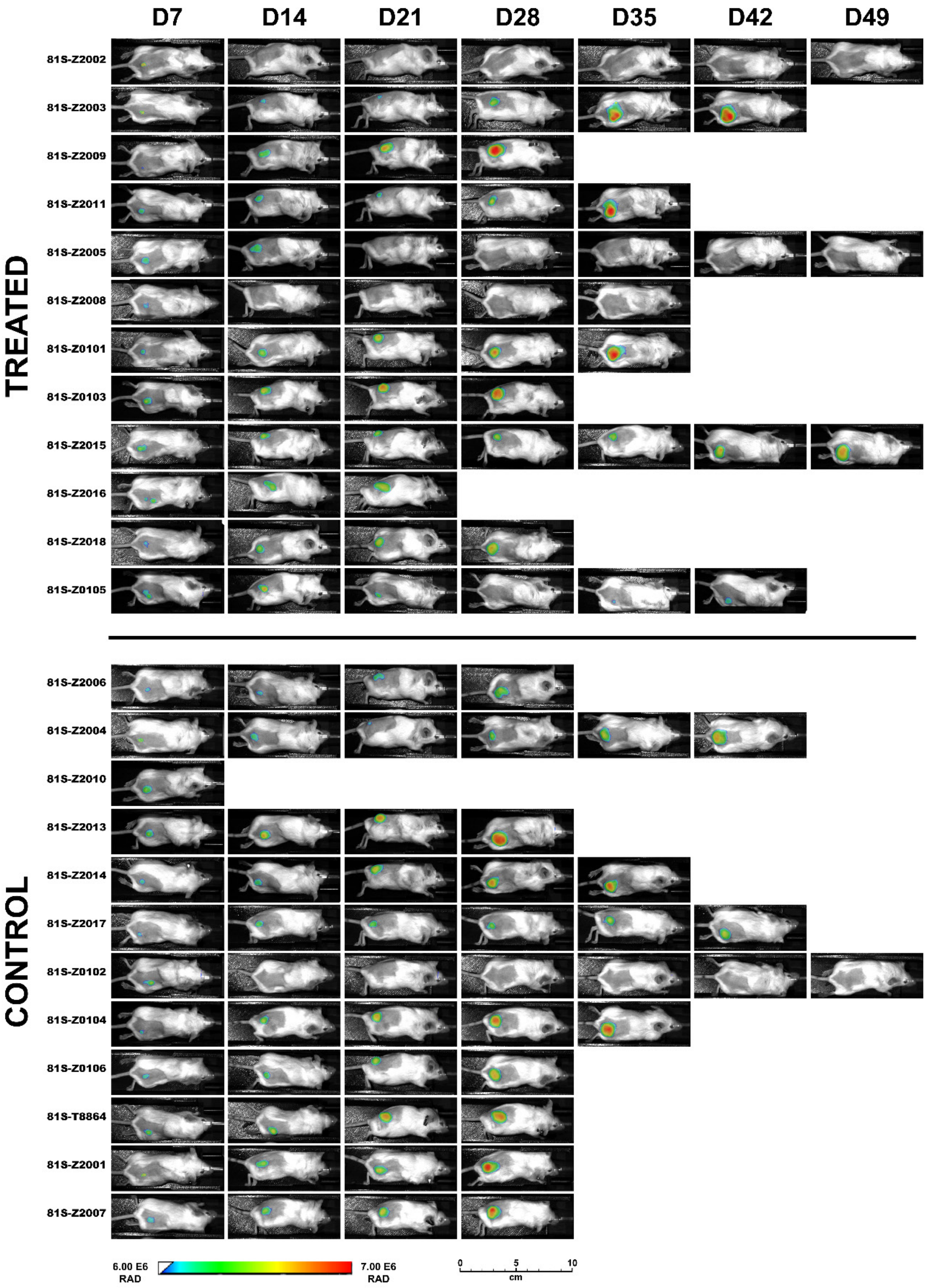

Figure S11. *In vivo* imaging of A20 lymphoma cells growth subcutaneously injected in syngeneic BALB/c mice.

A20 cell line was first modified to express mCardinal fluorescent protein. Five million of A20 tumor cells were injected subcutaneously D0 into the back of BALB/c mice and growth of tumors imaged every other week according to the schematics in Fig. 2C.

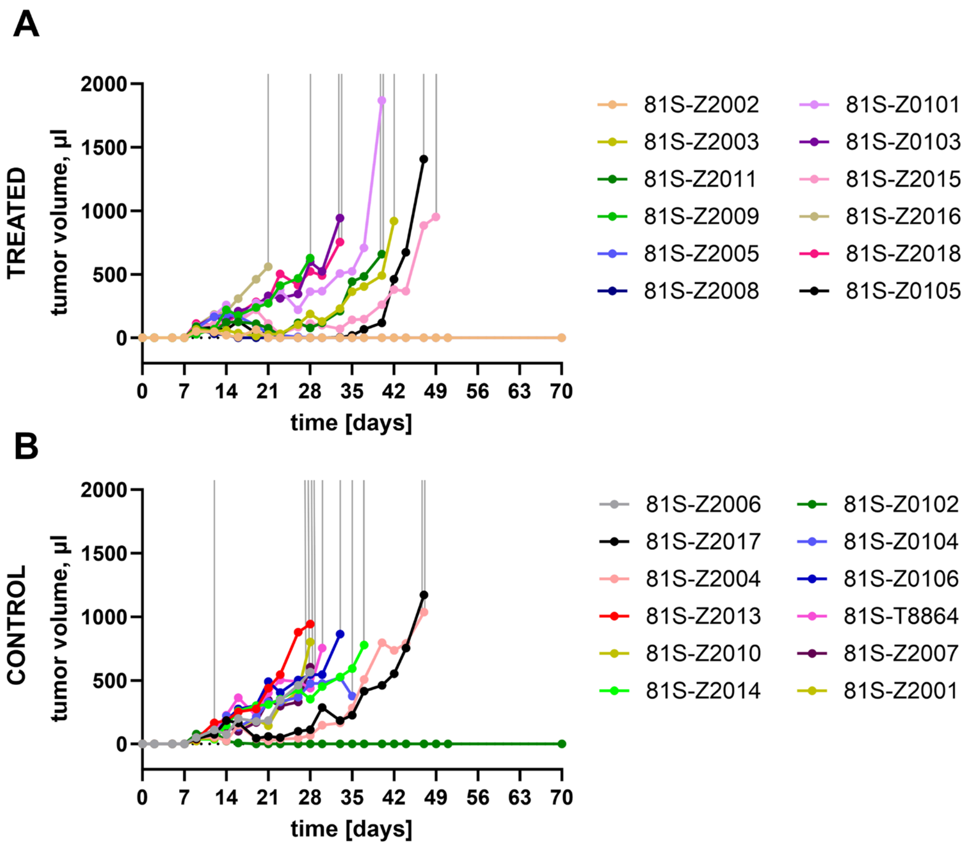

Figure S12. *In vivo* growth of A20 lymphoma cells subcutaneously injected in syngeneic BALB/c mice as measured by caliper.

Five million of A20 tumor cells were injected subcutaneously D0 into the back of BALB/c mice and tumor size measured three times per week in three dimensions by caliper. Gray lines depict times individual mice were sacrificed (at the time when at least one tumor diameter reached 1.5 cm). One mouse in the treated group died for unknown reasons, after successful treatment of the tumor without any signs of tumor growth. One mouse in the control group died, also for unknown reasons, with growing tumor before it reached 1.5. cm.

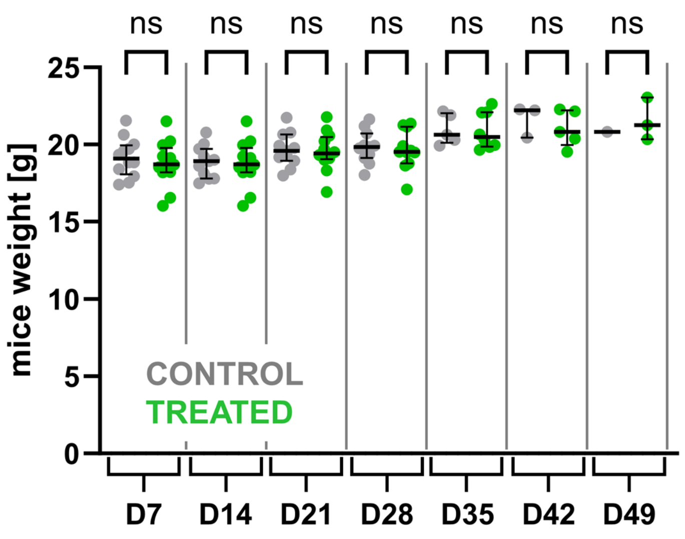

Figure S13. Treatment of BALB/c mice with selected combination of inhibitors did not affect mouse weight.

Treatment was not associated with any loss of weight. Mean with standard deviation are displayed. Ordinary one-way ANOVA with multiple comparisons.

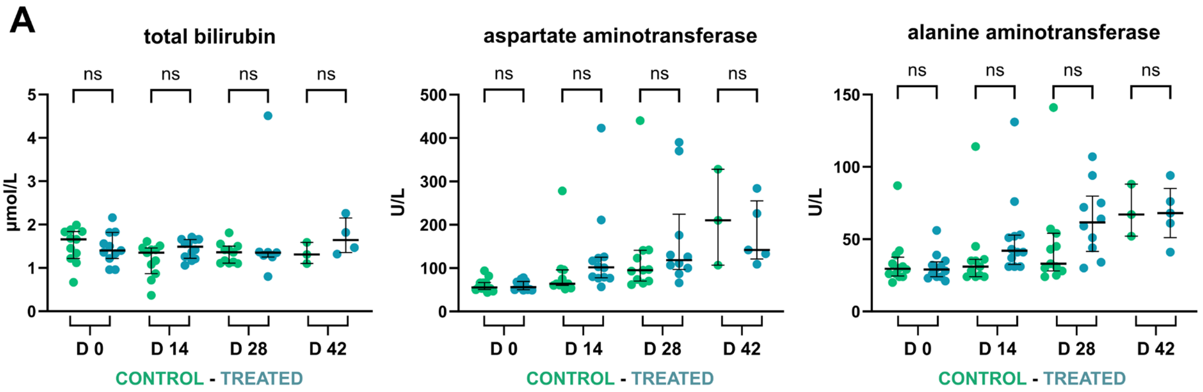

Parameter	Total Bilirubin	Aspartate aminotransferase	Alanine aminotransferase
Unit	mmol/L	U/L	U/L
C57Bl/6NCrl Mouse Blood Reference range	0.91-5.13	35.00-125.00	15.05-66.73

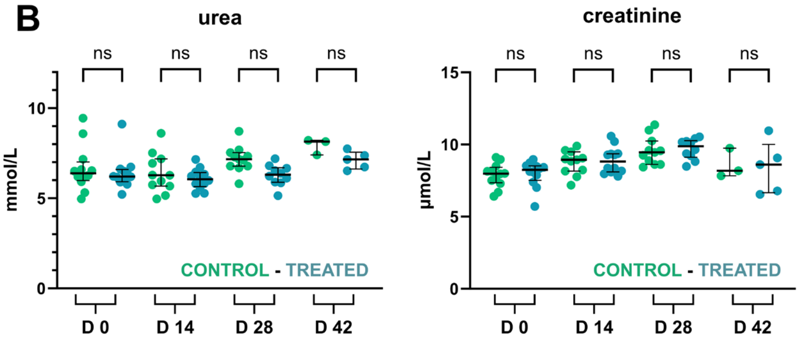

Parameter	Urea	Creatinine
Unit	mmol/L	mmol/L
C57Bl/6NCrl Mouse Blood Reference range	5.31-11.42	5.37-10.36

Figure S14. Treatment of BALB/c mice with selected combination of inhibitors did not affect liver and renal function.

Mice were treated with the combination of idelalisib, GSK2334470, and ipatasertib according to schematics in Fig. 2C. Blood was taken before experiment initiation and then every other week. There was no significant difference between treated and control group regarding **(A)** liver function (total bilirubin, aspartate aminotransferase, and alanine aminotransferase) and **(B)** renal function (urea and creatinine). Median with interquartile range is displayed. Kruskal-Wallis test with multiple comparisons. Tables show reference values.

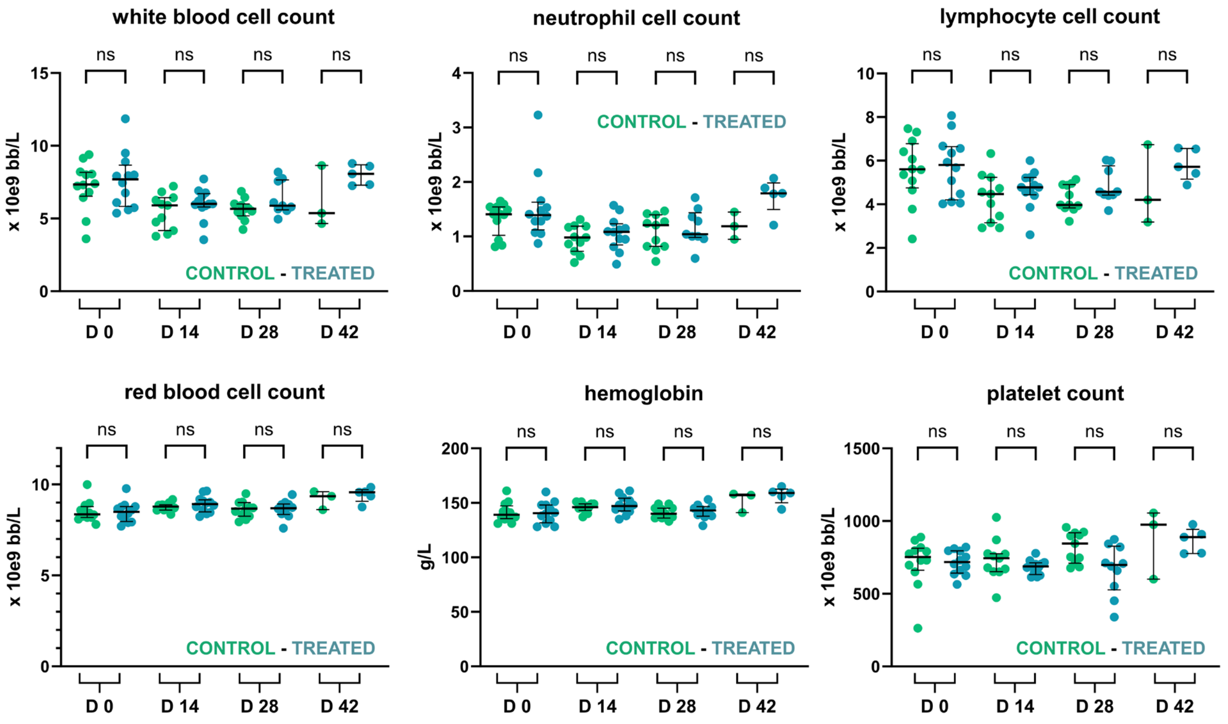

Parameter	White blood cell count	Neutrophil cell count	Lymphocyte cell count	Red blood cell count	Hemoglobin	Platelet count
Unit	x 10^9/L	x 10^9/L	x 10^9/L	x 10^{12}/L	g/L	x 10^9/L
C57Bl/6NCrl Mouse Blood Reference range	3.90-13.96	0.42-3.09	2.88-11.15	7.14-12.20	108-192	565-2159

Figure S15. Treatment of BALB/c mice with selected combination of inhibitors did not affect hematopoiesis.

Mice were treated with combination of idelalisib, GSK2334470, and ipatasertib according to schematics in Fig. 2C. Blood was taken before experiment initiation and then every other week. There was no significant difference between treated and control group regarding blood cell count (white blood cells, neutrophils, lymphocytes, red blood cells, hemoglobin, platelets). Median interquartile range is displayed. Kruskal-Wallis test with multiple comparisons. Table shows reference values.

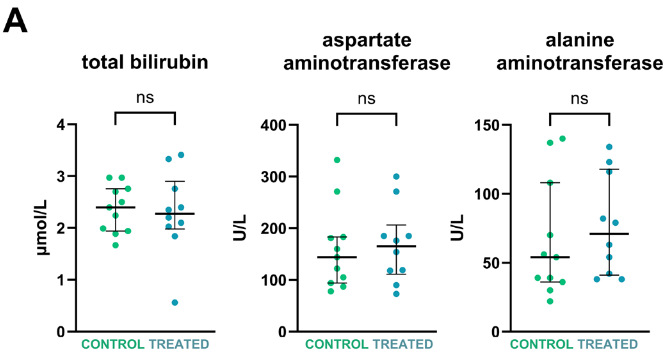

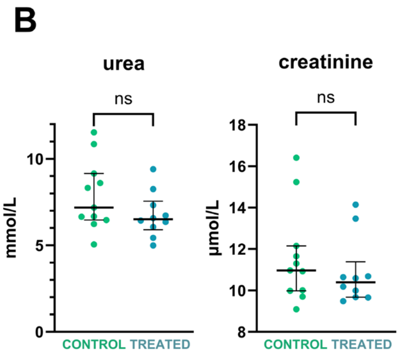

Parameter	Total Bilirubin	Aspartate aminotransferase	Alanine aminotransferase
Unit	mmol/L	U/L	U/L
C57Bl/6NCrl Mouse Blood Reference range	0.91-5.13	35.00-125.00	15.05-66.73

Parameter	Urea	Creatinine
Unit	mmol/L	mmol/L
C57Bl/6NCrl Mouse Blood Reference range	5.31-11.42	5.37-10.36

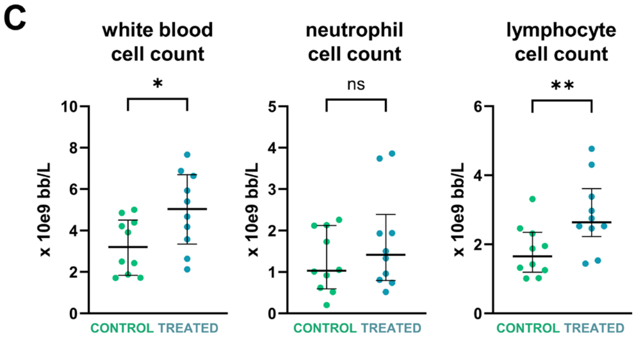

Parameter	White blood cell count	Neutrophil cell count	Lymphocyte cell count
Unit	x 10^9/L	x 10^9/L	x 10^9/L
C57Bl/6NCrl Mouse Blood Reference range	3.90-13.96	0.42-3.09	2.88-11.15

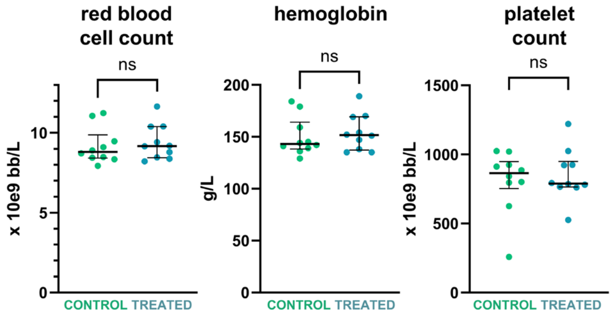

Parameter	Red blood cell count	Hemoglobin	Platelet count
Unit	x 10^{12}/L	g/L	x 10^9/L
C57Bl/6NCrl Mouse Blood Reference range	7.14-12.20	108-192	565-2159

Figure S16. Treatment of BALB/c mice with selected combination of inhibitors did not affect liver and real function at the time of mice sacrifice.

Mice were treated with combination of idelalisib, GSK2334470, and ipatasertib according to schematics in Fig. 2C. Blood was taken every other week and also at the time mice were sacrificed (displayed in this figure). There was no significant difference between treated and control group regarding **(A)** liver function (total bilirubin, aspartate aminotransferase, and alanine aminotransferase), **(B)** renal function (urea and creatinine), and **(C)** blood cell count (white blood cells, neutrophils, lymphocytes, red blood cells, hemoglobin, platelets). Median interquartile range is displayed. Kruskal-Wallis test with multiple comparisons. Tables show reference values.

SUPPLEMENTARY REFERENCES

1. Havranek O, Xu J, Koehrer S, Wang Z, Becker L, Comer JM, et al. Tonic B-cell receptor signaling in diffuse large B-cell lymphoma. Blood. 2017;130(8):995-1006.
2. Kowarz E, Loscher D, Marschalek R. Optimized Sleeping Beauty transposons rapidly generate stable transgenic cell lines. Biotechnology journal. 2015;10(4):647-53.
3. Henderson J, Havranek O, Ma MCJ, Herman V, Kupcova K, Chrbolkova T, et al. Detecting Förster resonance energy transfer in living cells by conventional and spectral flow cytometry. Cytometry Part A : the journal of the International Society for Analytical Cytology. 2022;101(10):818-34.
4. Mates L, Chuah MK, Belay E, Jerchow B, Manoj N, Acosta-Sanchez A, et al. Molecular evolution of a novel hyperactive Sleeping Beauty transposase enables robust stable gene transfer in vertebrates. Nature genetics. 2009;41(6):753-61.
5. Chu J, Haynes RD, Corbel SY, Li P, González-González E, Burg JS, et al. Non-invasive intravital imaging of cellular differentiation with a bright red-excitable fluorescent protein. Nat Methods. 2014;11(5):572-8.
6. Zheng S, Wang W, Aldahdooh J, Malyutina A, Shadbahr T, Tanoli Z, et al. SynergyFinder Plus: Toward Better Interpretation and Annotation of Drug Combination Screening Datasets. Genomics Proteomics Bioinformatics. 2022;20(3):587-96.
7. Yadav B, Wennerberg K, Aittokallio T, Tang J. Searching for Drug Synergy in Complex Dose-Response Landscapes Using an Interaction Potency Model. Computational and structural biotechnology journal. 2015;13:504-13.
8. Kim KJ, Kanellopoulos-Langevin C, Merwin RM, Sachs DH, Asofsky R. Establishment and characterization of BALB/c lymphoma lines with B cell properties. Journal of immunology. 1979;122(2):549-54.
9. Maharaj K, Powers JJ, Achille A, Mediavilla-Varela M, Gamal W, Burger KL, et al. The dual PI3Kδ/CK1ε inhibitor umbralisib exhibits unique immunomodulatory effects on CLL T cells. Blood Adv. 2020;4(13):3072-84.
10. Yang C, Huang X, Liu H, Xiao F, Wei J, You L, et al. PDK1 inhibitor GSK2334470 exerts antitumor activity in multiple myeloma and forms a novel multitargeted combination with dual mTORC1/C2 inhibitor PP242. Oncotarget. 2017;8(24):39185-97.
11. Sun L, Huang Y, Liu Y, Zhao Y, He X, Zhang L, et al. Ipatasertib, a novel Akt inhibitor, induces transcription factor FoxO3a and NF-κB directly regulates PUMA-dependent apoptosis. Cell Death Dis. 2018;9(9):911.
12. Nair AB, Jacob S. A simple practice guide for dose conversion between animals and human. J Basic Clin Pharm. 2016;7(2):27-31.
