# Supplementary material for: Vertical targeting of the PI3K/AKT pathway at multiple points is synergistic and effective for non-Hodgkin lymphoma
Source: Exp Hematol Oncol. 2024 Nov 1;13:108. doi: 10.1186/s40164-024-00568-6 (PMC11529427; doi:10.1186/s40164-024-00568-6)
Supplement: Supplementary file 2 — Supplementary Material 2 [file 40164_2024_568_MOESM2_ESM.pdf]

SUPPLEMENTARY TABLES

Vertical Targeting of the PI3K/AKT Pathway at Multiple Points is Synergistic and Effective for Non- Hodgkin Lymphoma

Kristyna Kupcova[1,2], Jana Senavova[1,2], Filip Jura[1], Vaclav Herman[1,2], Anezka Rajmonova[1], Mariana Pacheco-Blanco[1], Tereza Chrbolkova[1], Iva Hamova[1,2], R. Eric Davis[3], and Ondrej Havranek[1,2]

[1] BIOCEV, First Faculty of Medicine, Charles University, Prague, Czech Republic

[2] First Department of Medicine – Department of Hematology, First Faculty of Medicine, Charles University and General University Hospital, Prague, Czech Republic

[3] Department of Lymphoma and Myeloma, The University of Texas MD Anderson Cancer Center, Houston, TX, U.S.A.

Correspondence: Ondrej Havranek, BIOCEV, First Faculty of Medicine, Charles University, Prumyslova 595, 25250, Czech Republic, +420 325873029, ondrej.havranek@lf1.cuni.cz

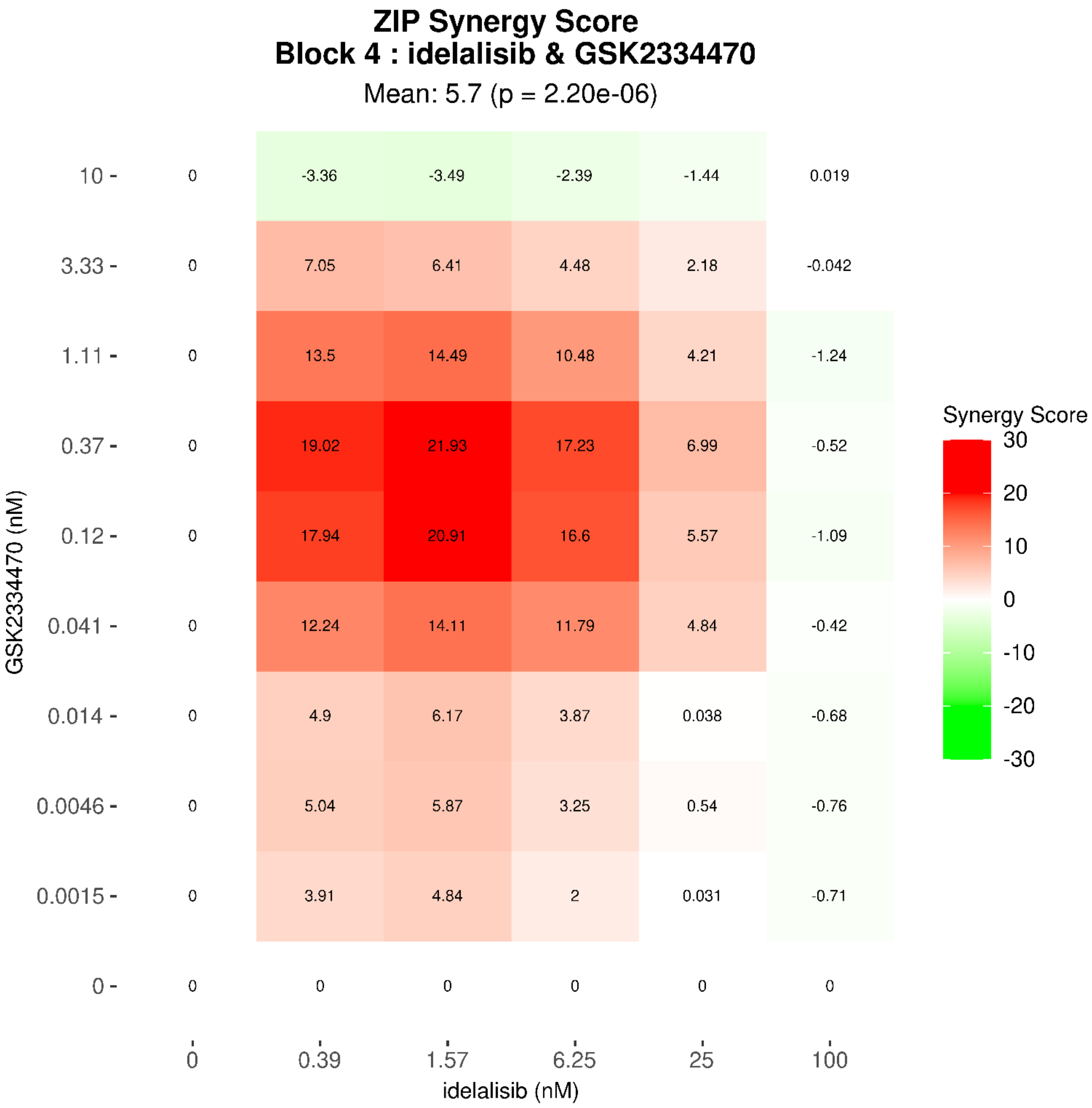

Table S1. Synergy scores for pairwise combination of idelalisib and GSK2334470 in OCI-Ly7 cells displayed in Fig. 2A (first replicate). Viable cell numbers were measured after 96 hours incubation. Synergy scores were calculation using zero interaction potency model (SynergyFinder3.0).

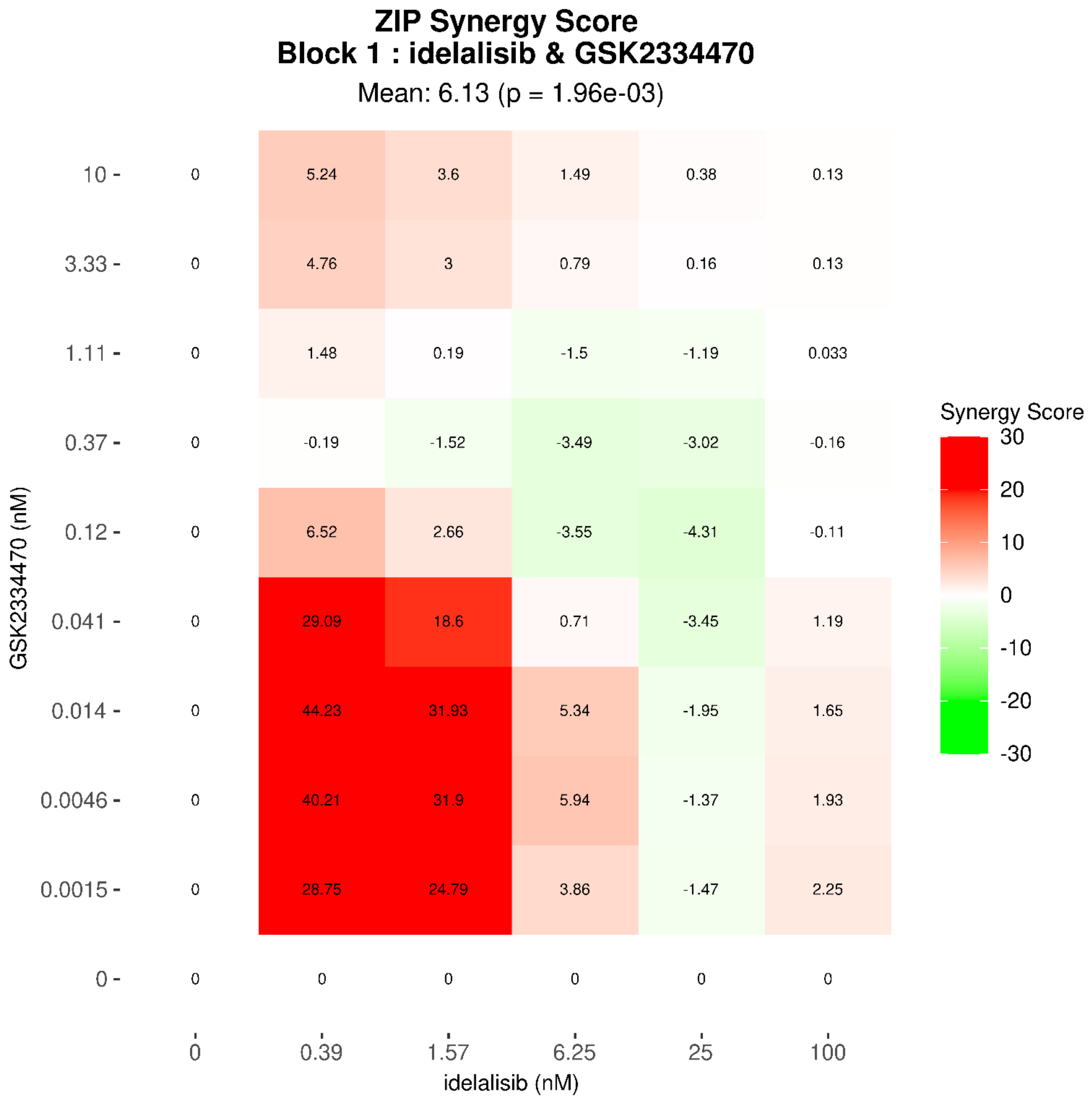

Table S2. Synergy scores for pairwise combination of idelalisib and GSK2334470 in SUDHL-4 cells displayed in Fig. 2A (first replicate). Viable cell numbers were measured after 96 hours incubation. Synergy scores were calculation using zero interaction potency model (SynergyFinder3.0).

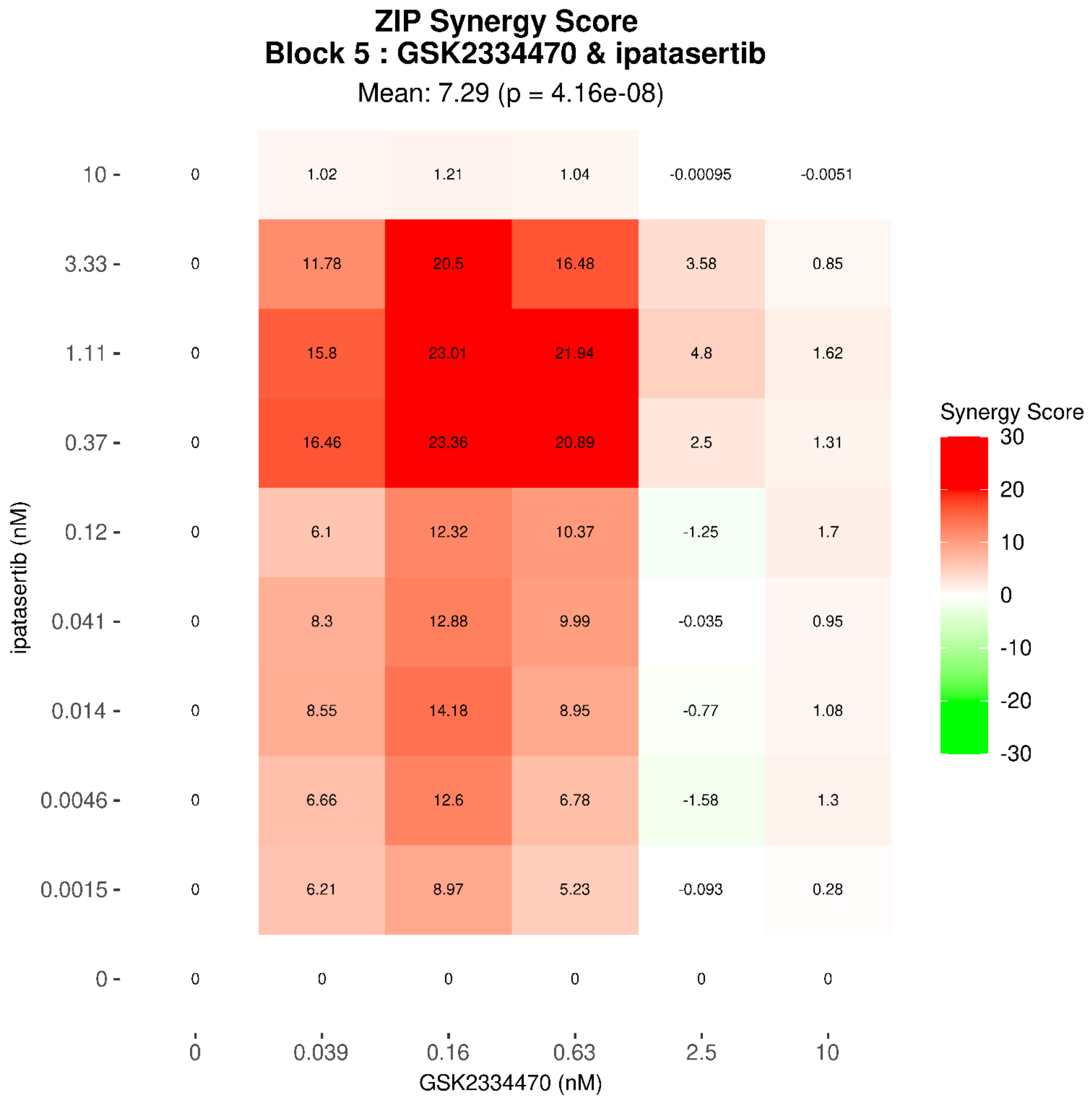

Table S3. Synergy scores for pairwise combination of GSK2334470 and ipatasertib in OCI-Ly7 cells displayed in Fig. 2A (first replicate). Viable cell numbers were measured after 96 hours incubation. Synergy scores were calculation using zero interaction potency model (SynergyFinder3.0).

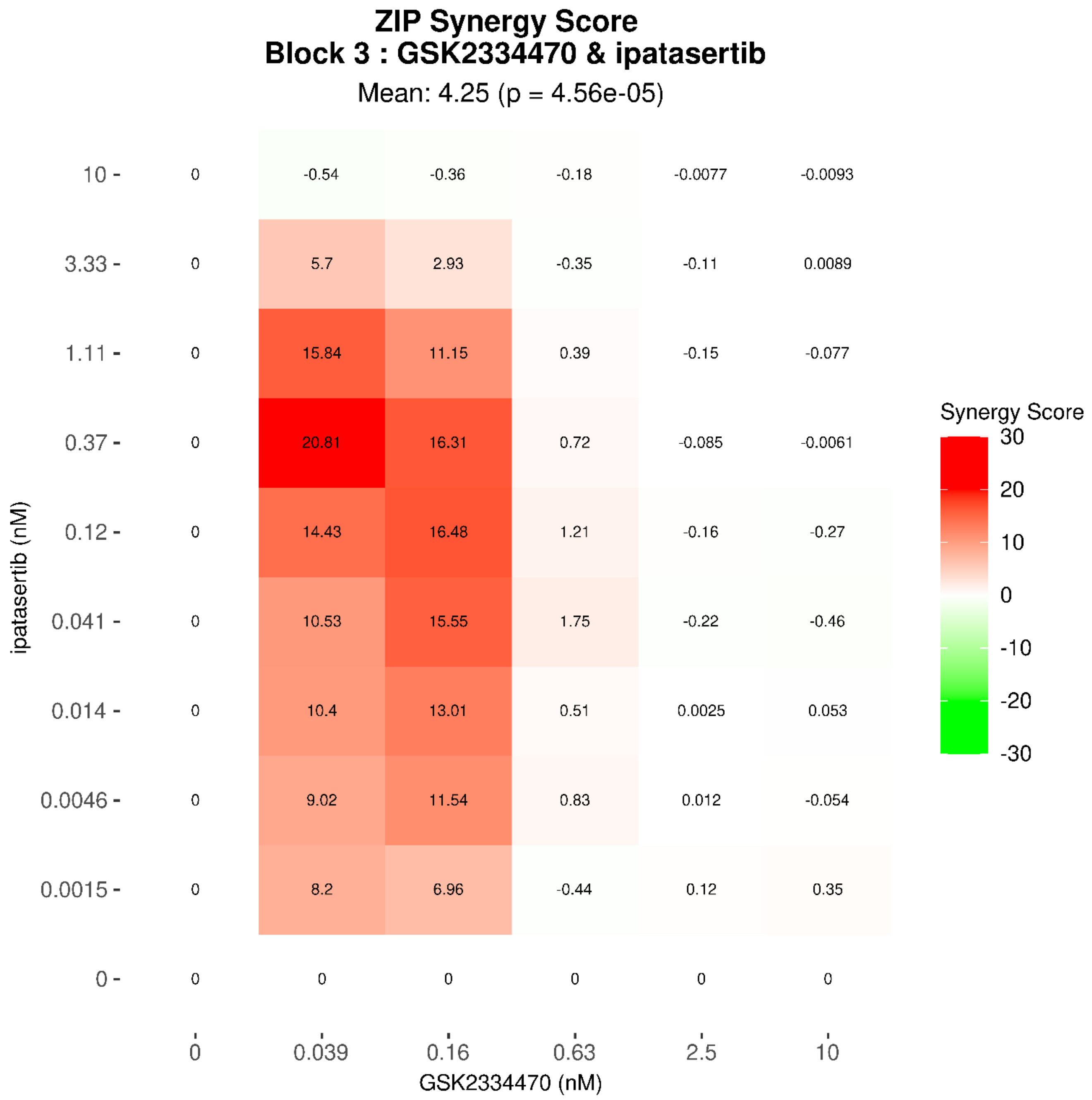

Table S4. Synergy scores for pairwise combination of GSK2334470 and ipatasertib in SUDHL-4 cells displayed in Fig. 2A (first replicate). Viable cell numbers were measured after 96 hours incubation. Synergy scores were calculation using zero interaction potency model (SynergyFinder3.0).

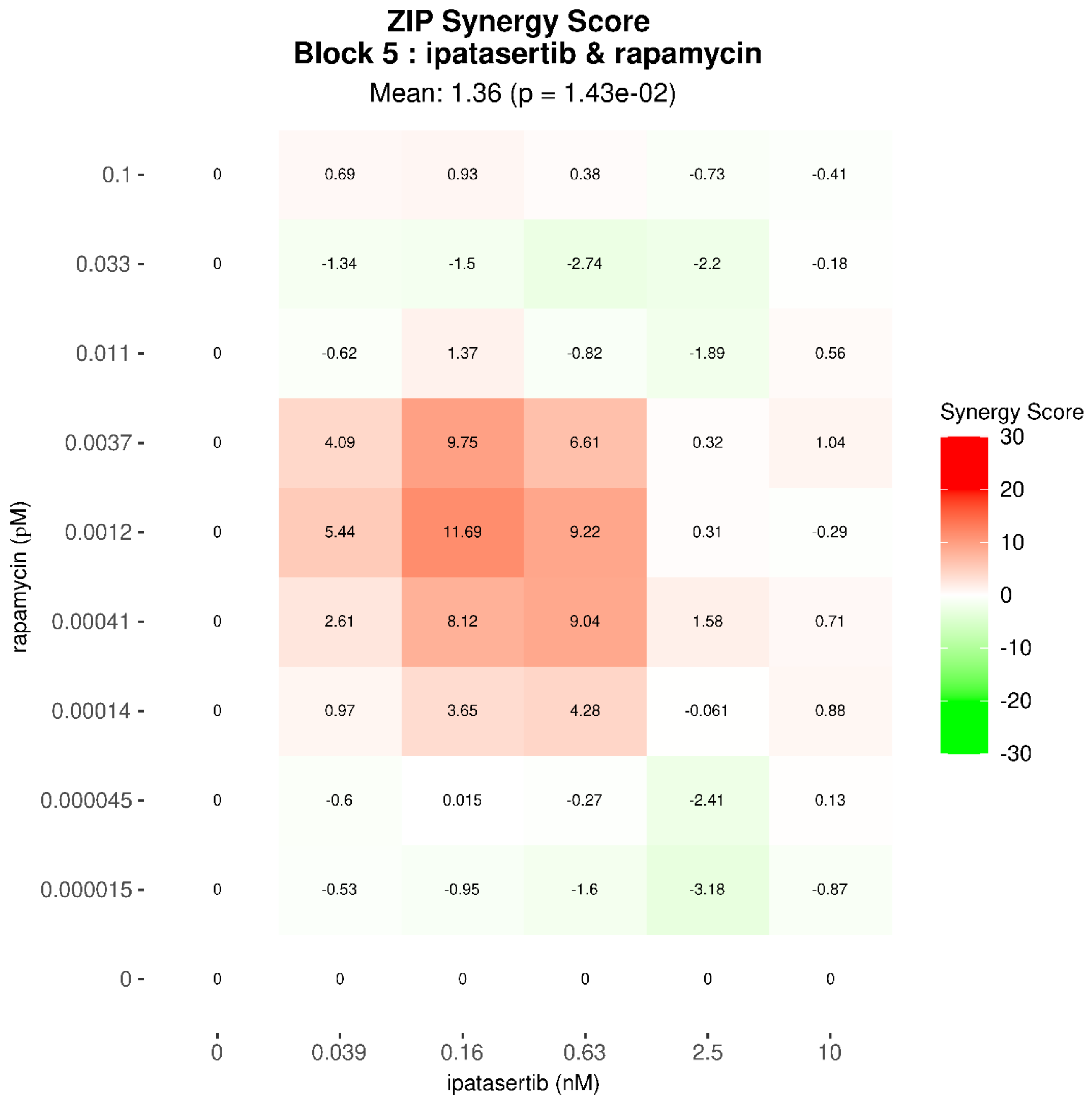

Table S5. Synergy scores for pairwise combination of ipatasertib and rapamycin in OCI-Ly7 cells displayed in Fig. 2A (first replicate). Viable cell numbers were measured after 96 hours incubation. Synergy scores were calculation using zero interaction potency model (SynergyFinder3.0).

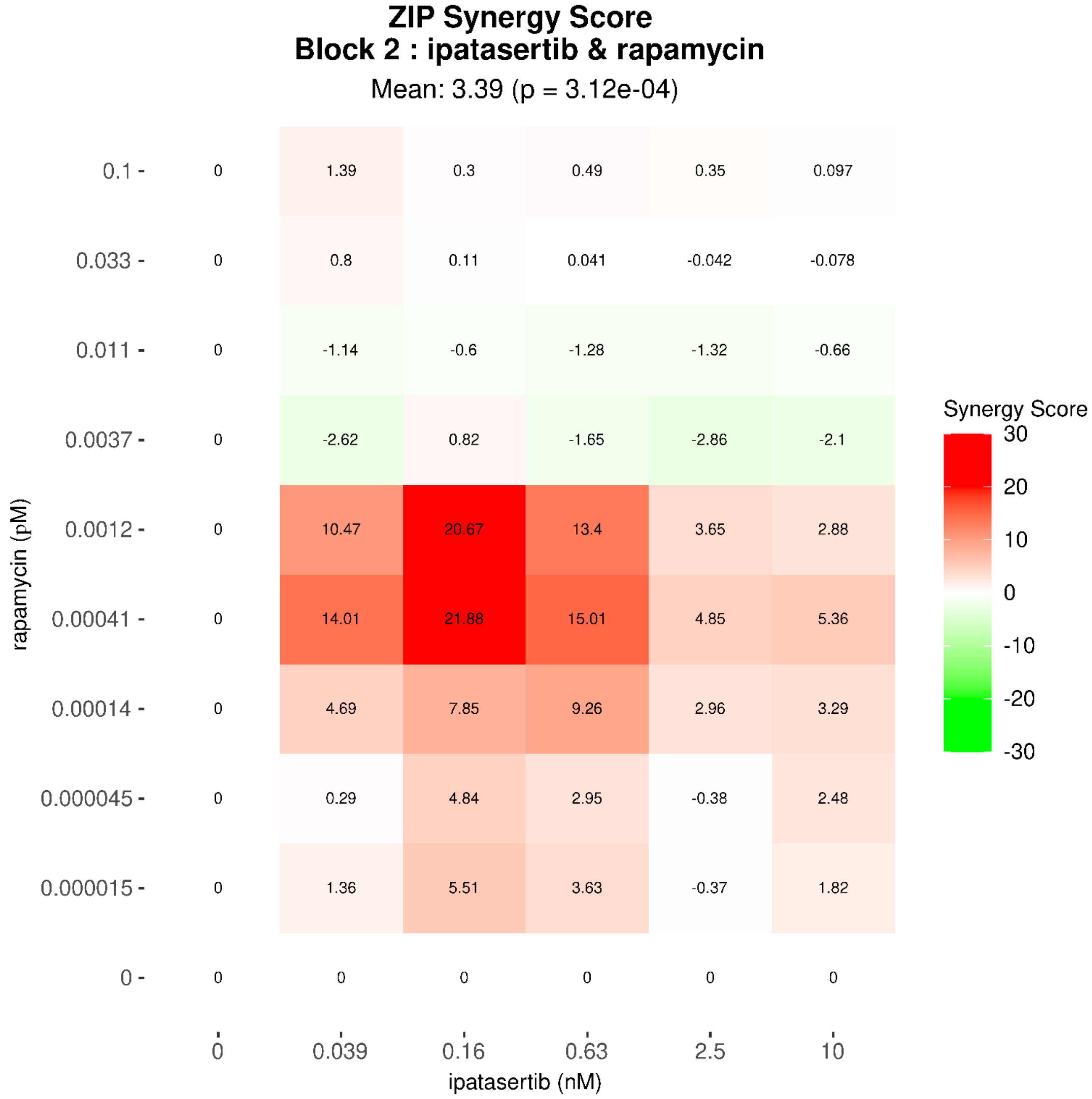

Table S6. Synergy scores for pairwise combination of ipatasertib and rapamycin in SUDHL-4 cells displayed in Fig. 2A (first replicate). Viable cell numbers were measured after 96 hours incubation. Synergy scores were calculation using zero interaction potency model (SynergyFinder3.0).

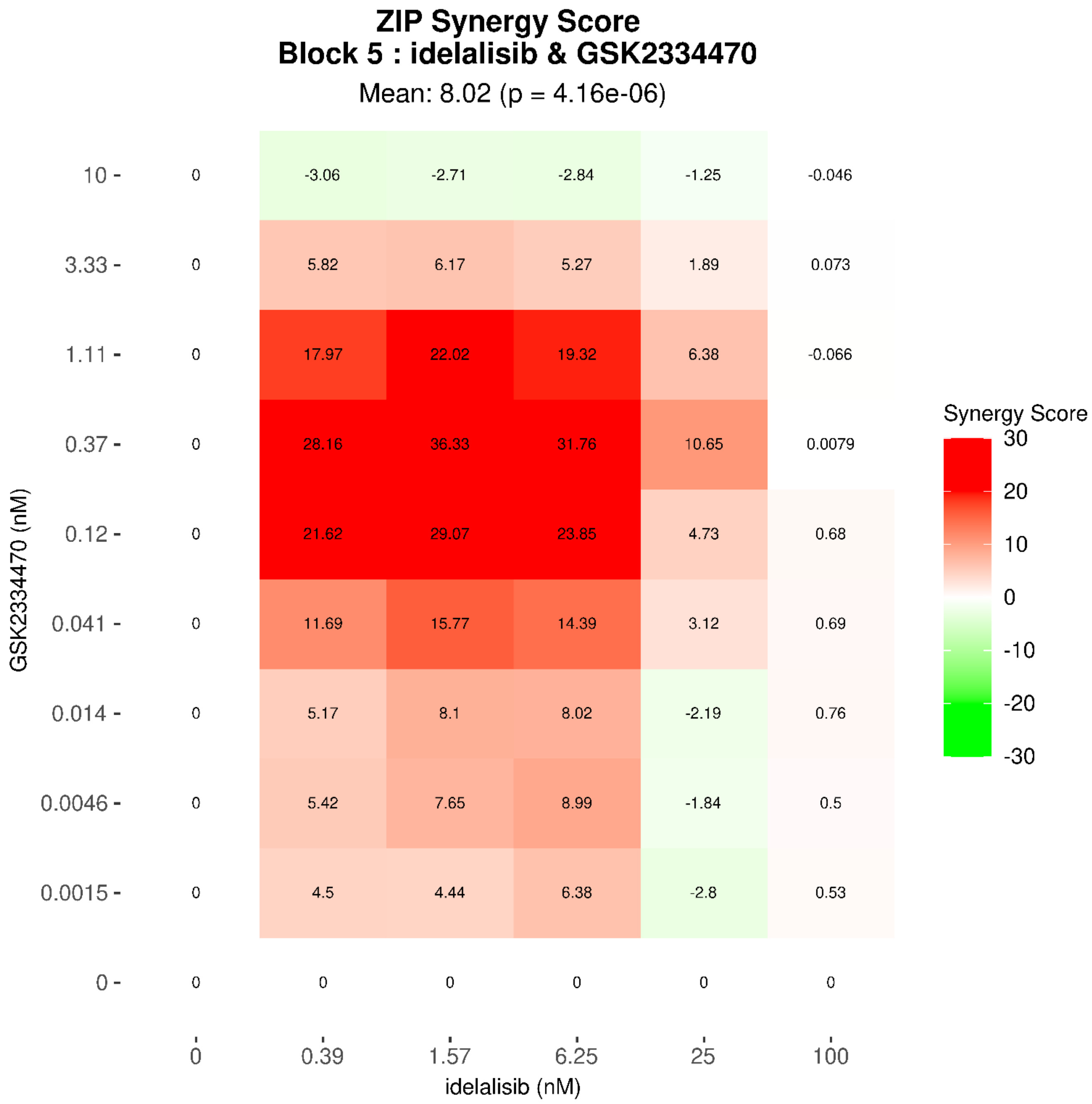

Table S7. Synergy scores for pairwise combination of idelalisib and GSK2334470 in OCI-Ly7 cells displayed in Fig. S6 (second replicate). Viable cell numbers were measured after 96 hours incubation. Synergy scores were calculation using zero interaction potency model (SynergyFinder3.0).

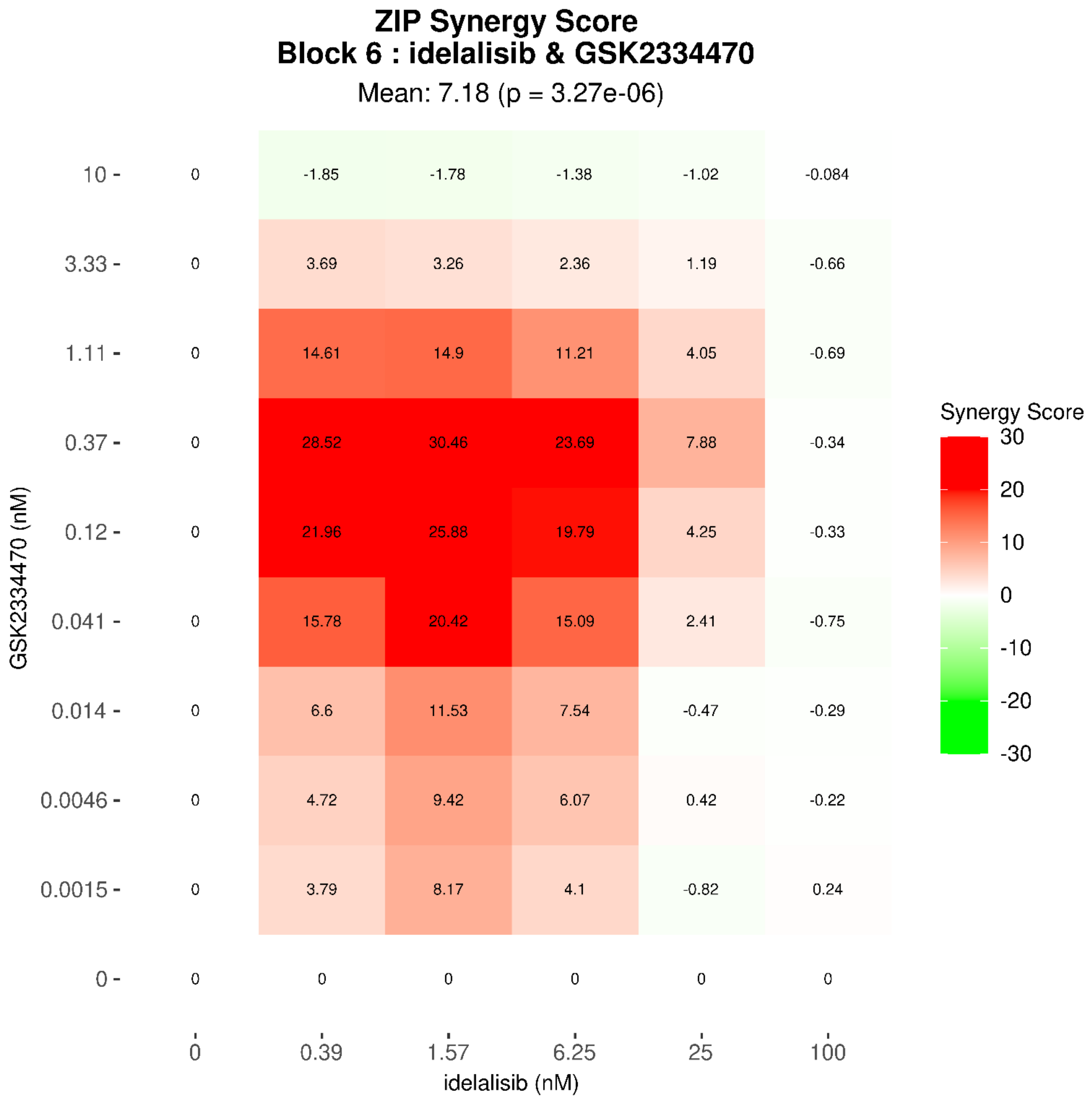

Table S8. Synergy scores for pairwise combination of idelalisib and GSK2334470 in OCI-Ly7 cells displayed in Fig. S6 (third replicate). Viable cell numbers were measured after 96 hours incubation. Synergy scores were calculation using zero interaction potency model (SynergyFinder3.0).

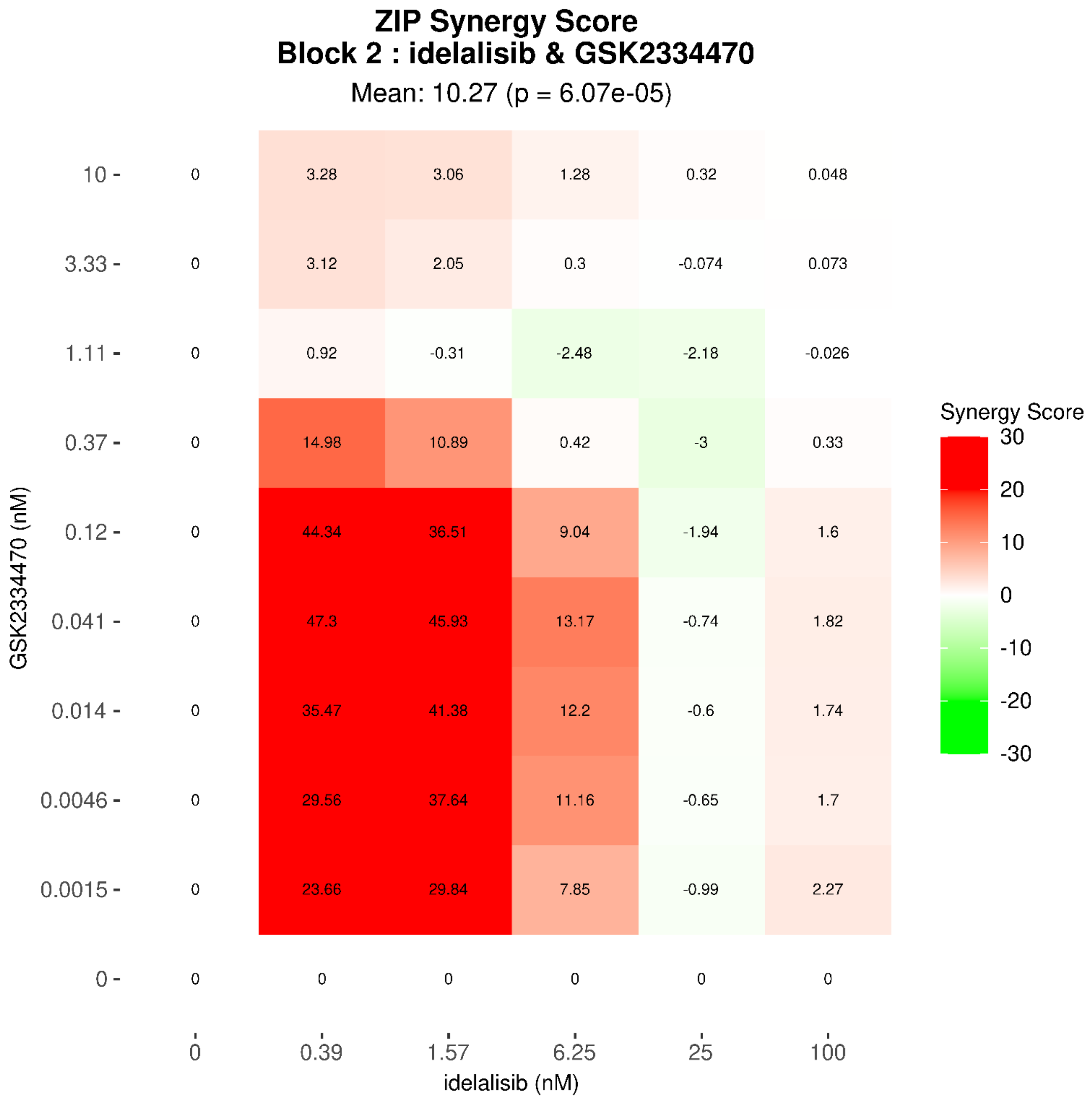

Table S9. Synergy scores for pairwise combination of idelalisib and GSK2334470 in SUDHL-4 cells displayed in Fig. S6 (second replicate). Viable cell numbers were measured after 96 hours incubation. Synergy scores were calculation using zero interaction potency model (SynergyFinder3.0).

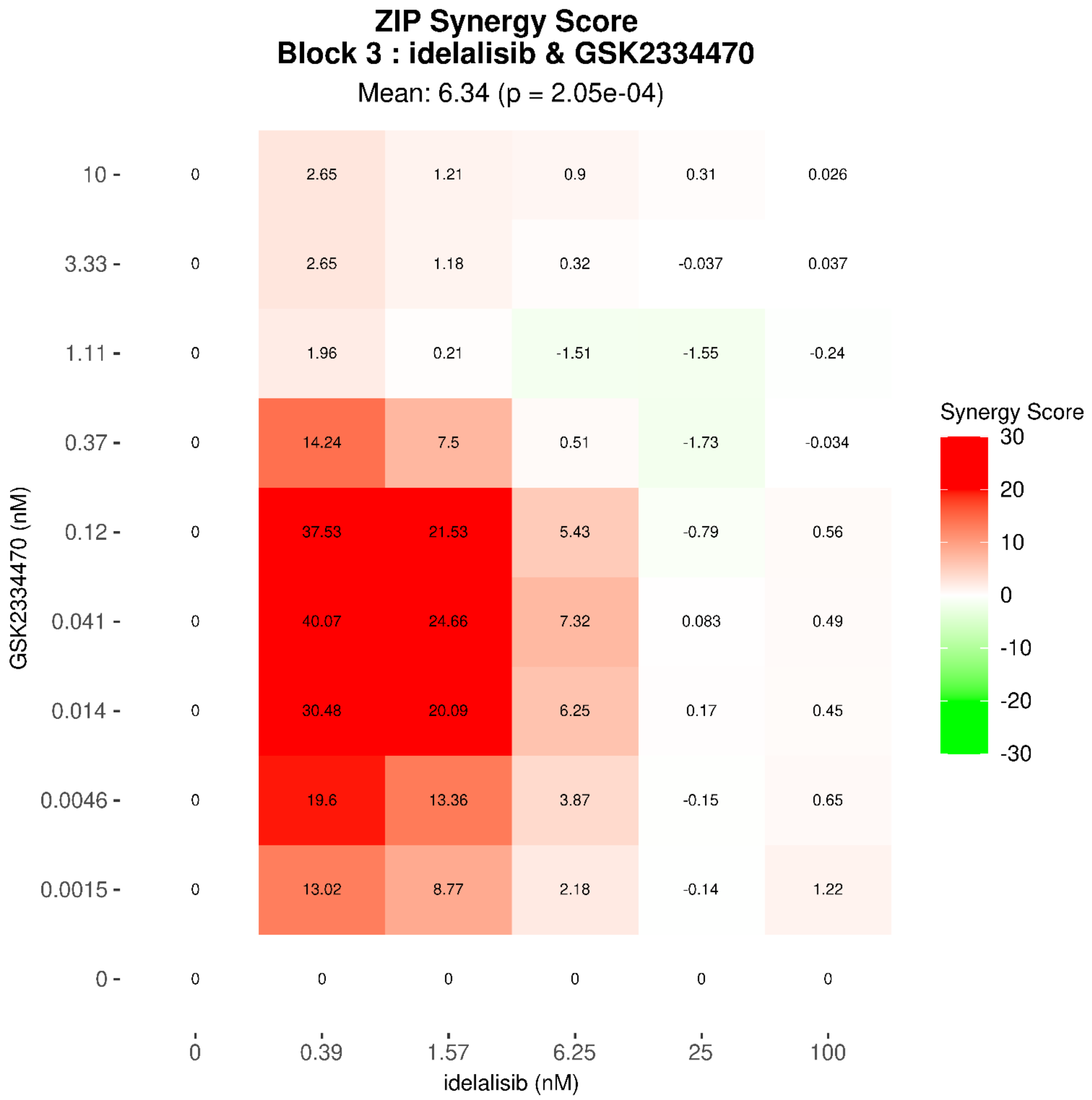

Table S10. Synergy scores for pairwise combination of idelalisib and GSK2334470 in SUDHL-4 cells displayed in Fig. S6 (third replicate). Viable cell numbers were measured after 96 hours incubation. Synergy scores were calculation using zero interaction potency model (SynergyFinder3.0).

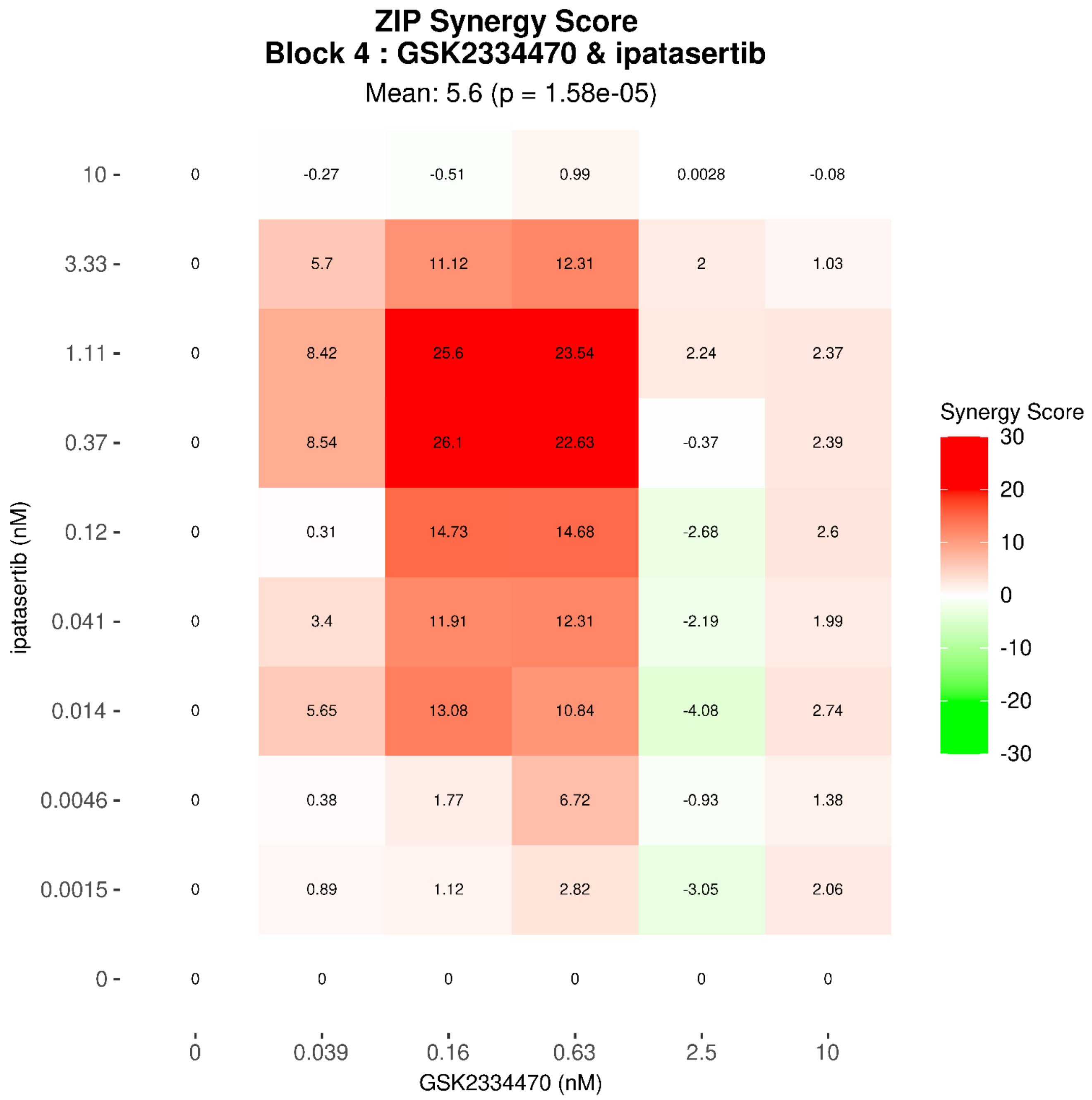

Table S11. Synergy scores for pairwise combination of GSK2334470 and ipatasertib in OCI-Ly7 cells displayed in Fig. S6 (second replicate). Viable cell numbers were measured after 96 hours incubation. Synergy scores were calculation using zero interaction potency model (SynergyFinder3.0).

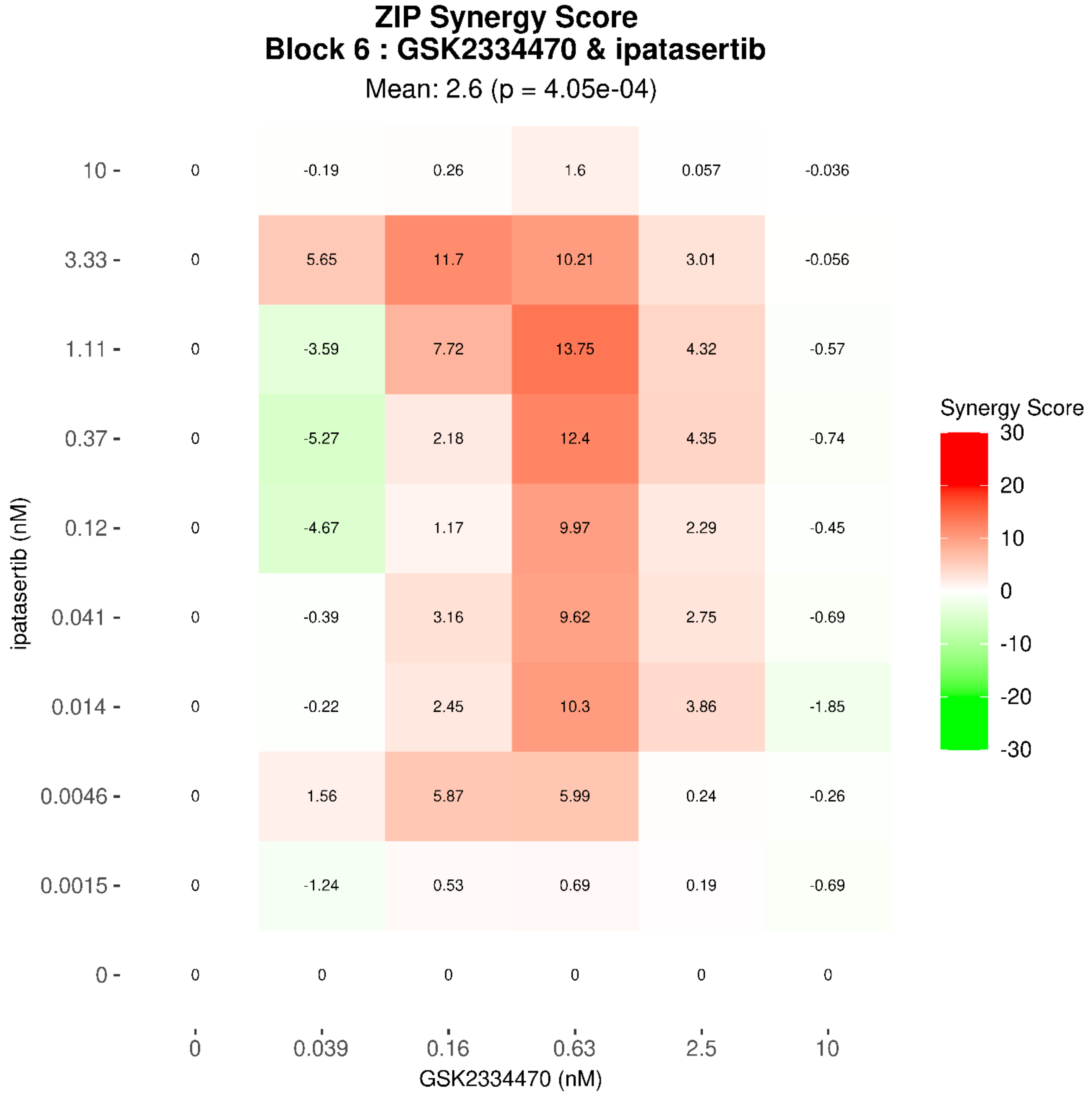

Table S12. Synergy scores for pairwise combination of GSK2334470 and ipatasertib in OCI-Ly7 cells displayed in Fig. S6 (third replicate). Viable cell numbers were measured after 96 hours incubation. Synergy scores were calculation using zero interaction potency model (SynergyFinder3.0).

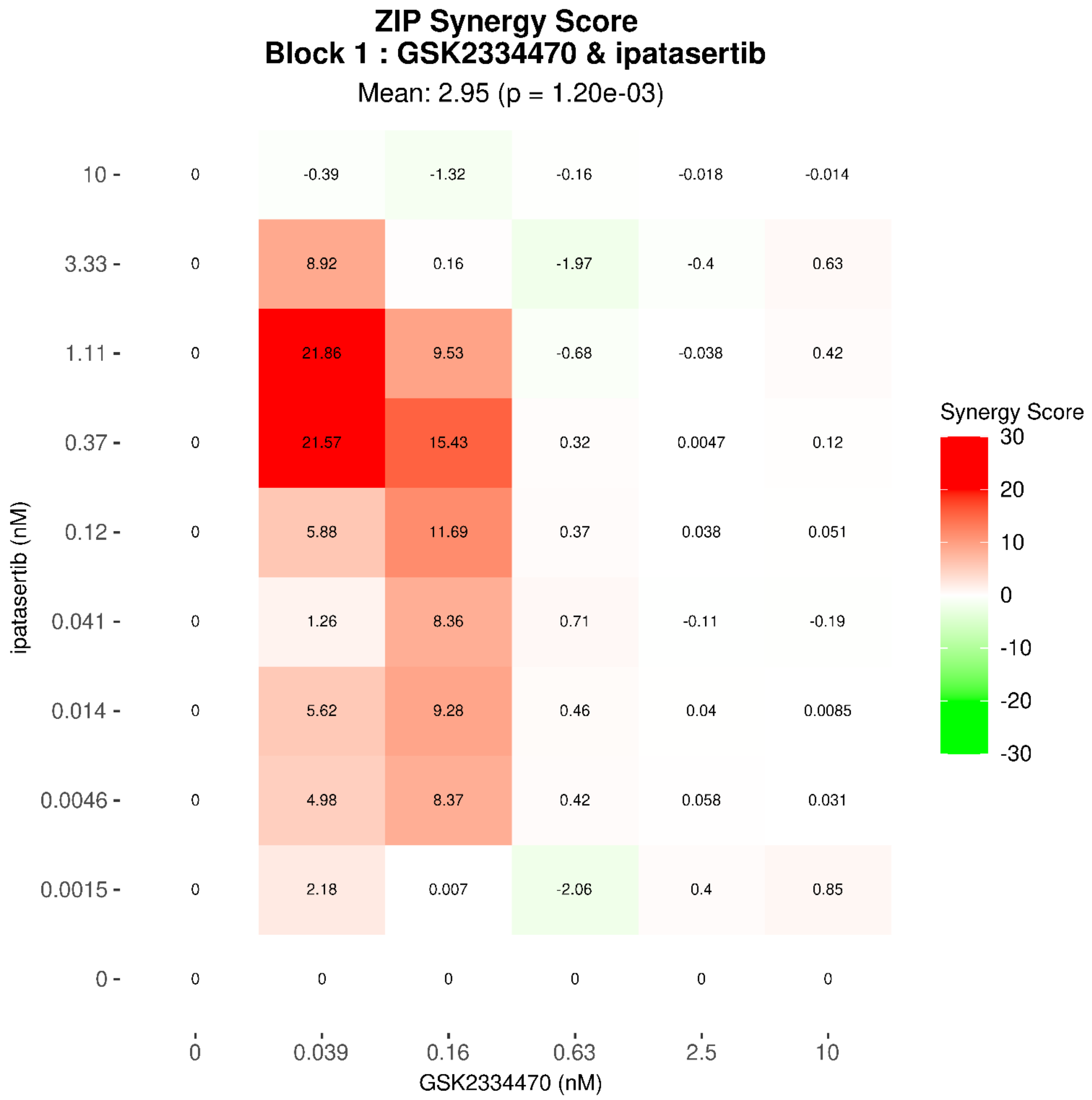

Table S13. Synergy scores for pairwise combination of GSK2334470 and ipatasertib in SUDHL-4 cells displayed in Fig. S6 (second replicate). Viable cell numbers were measured after 96 hours incubation. Synergy scores were calculation using zero interaction potency model (SynergyFinder3.0).

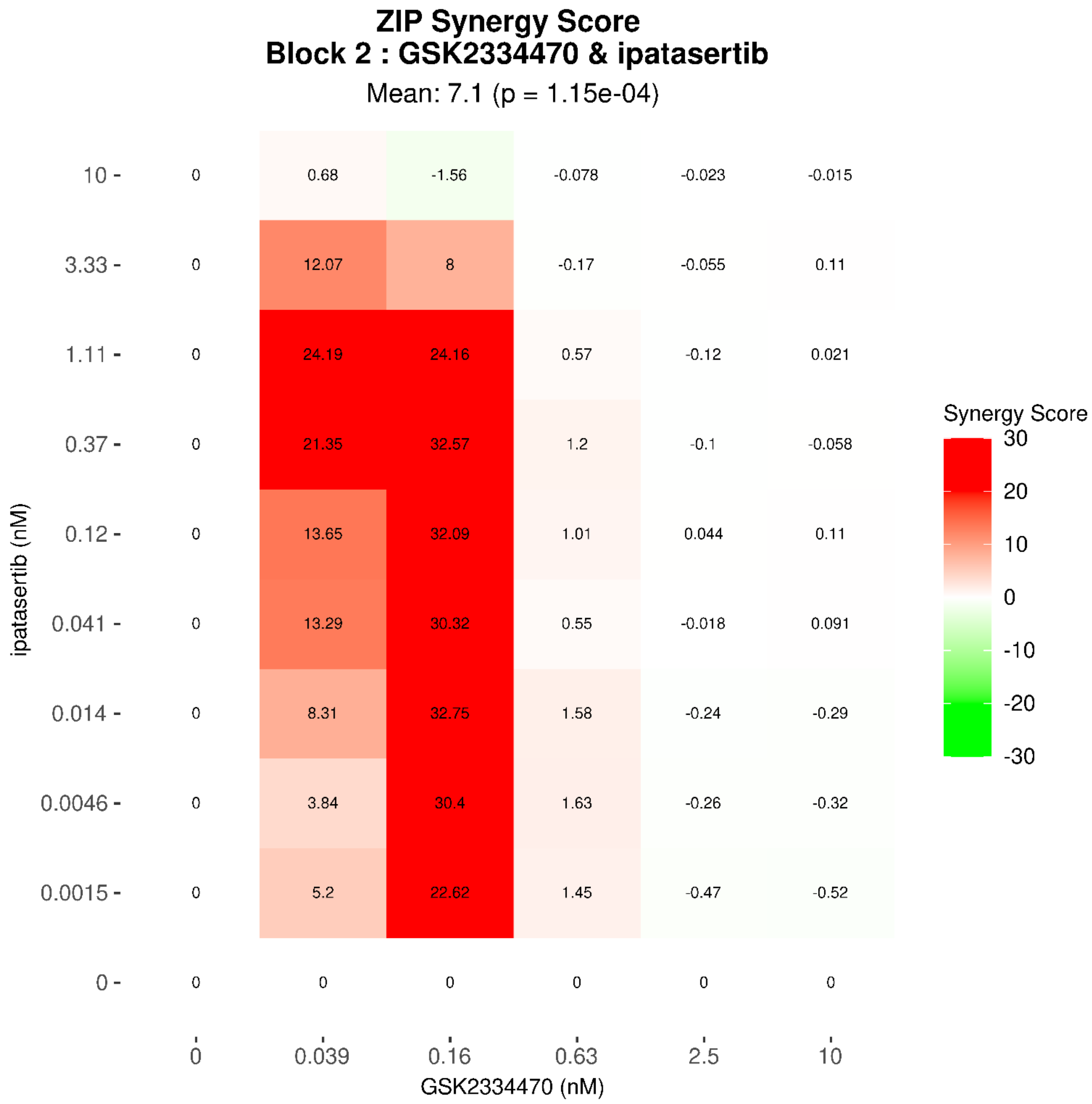

Table S14. Synergy scores for pairwise combination of GSK2334470 and ipatasertib in SUDHL-4 cells displayed in Fig. S6 (third replicate). Viable cell numbers were measured after 96 hours incubation. Synergy scores were calculation using zero interaction potency model (SynergyFinder3.0).

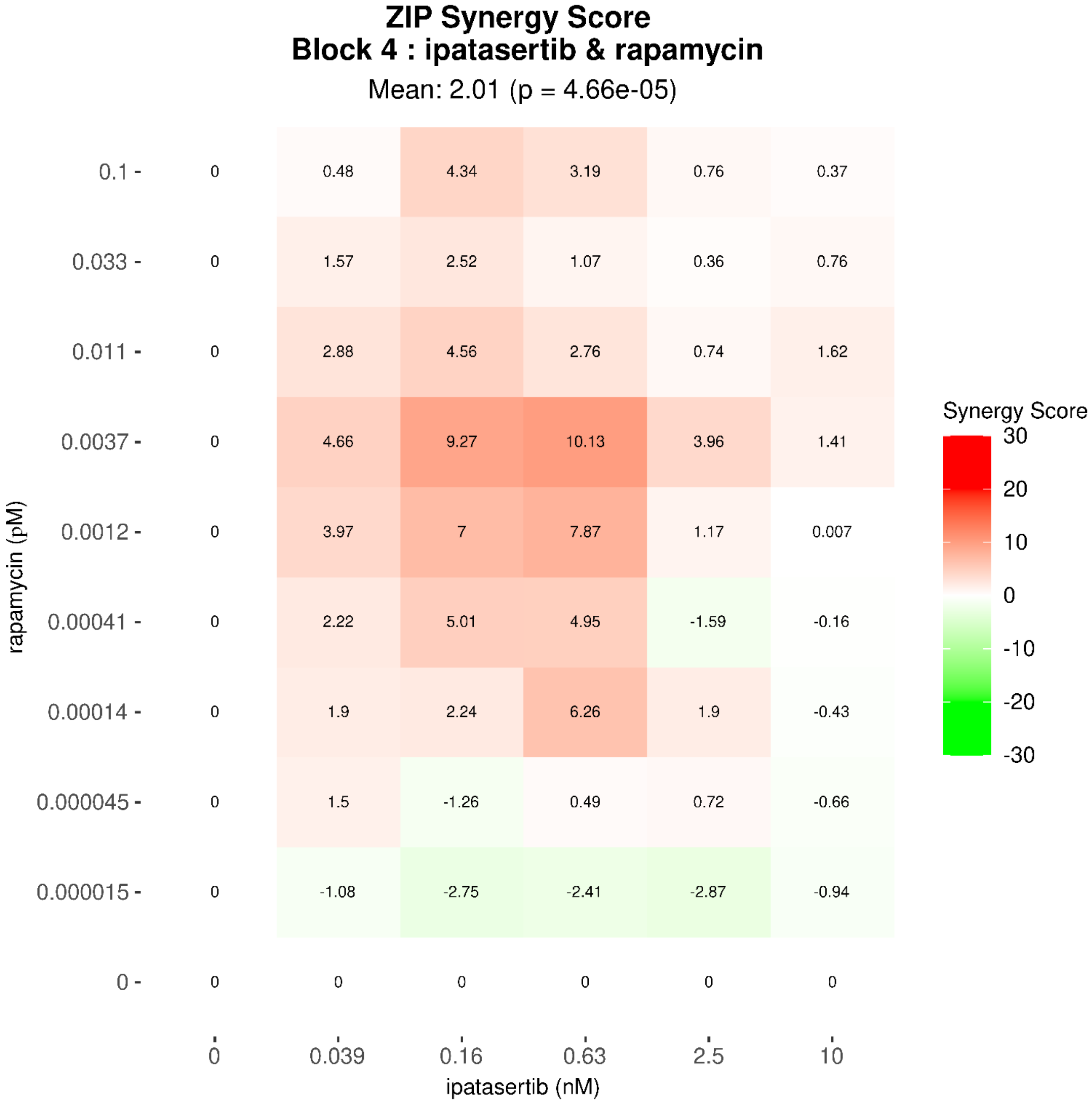

Table S15. Synergy scores for pairwise combination of ipatasertib and rapamycin in OCI-Ly7 cells displayed in Fig. S6 (second replicate). Viable cell numbers were measured after 96 hours incubation. Synergy scores were calculation using zero interaction potency model (SynergyFinder3.0).

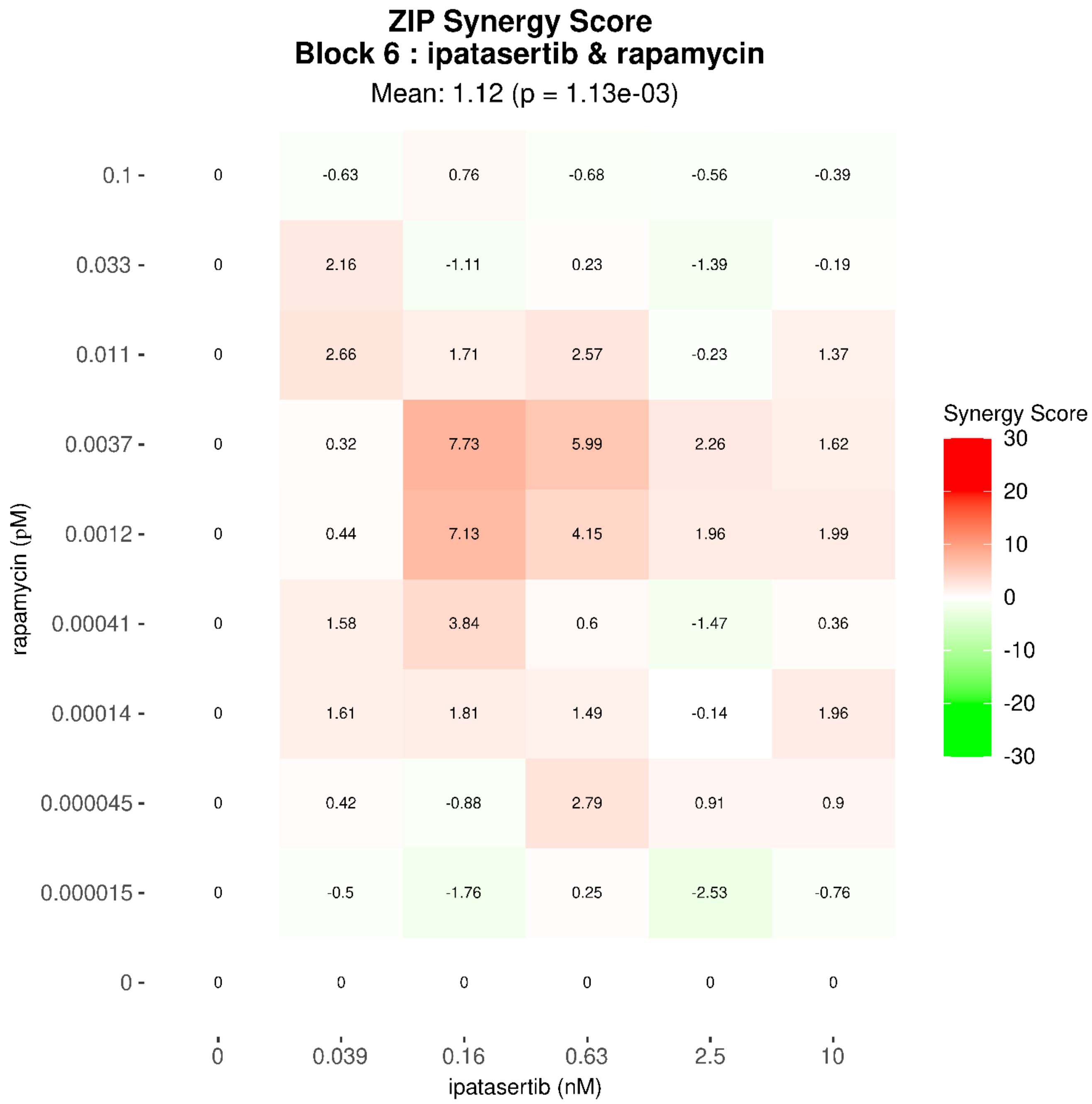

Table S16. Synergy scores for pairwise combination of ipatasertib and rapamycin in OCI-Ly7 cells displayed in Fig. S6 (third replicate). Viable cell numbers were measured after 96 hours incubation. Synergy scores were calculation using zero interaction potency model (SynergyFinder3.0).

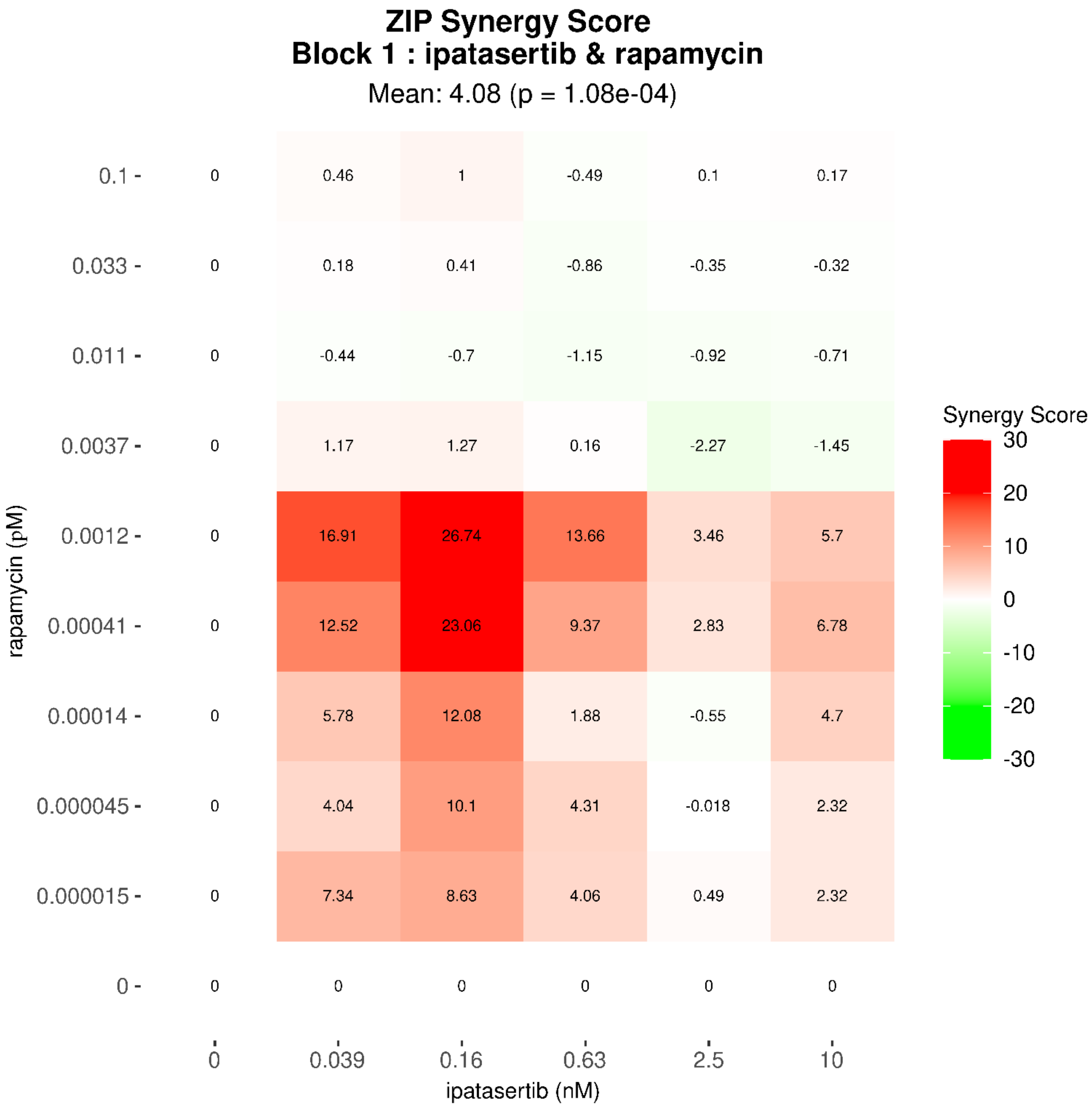

Table S17. Synergy scores for pairwise combination of ipatasertib and rapamycin in SUDHL-4 cells displayed in Fig. S6 (second replicate). Viable cell numbers were measured after 96 hours incubation. Synergy scores were calculation using zero interaction potency model (SynergyFinder3.0).

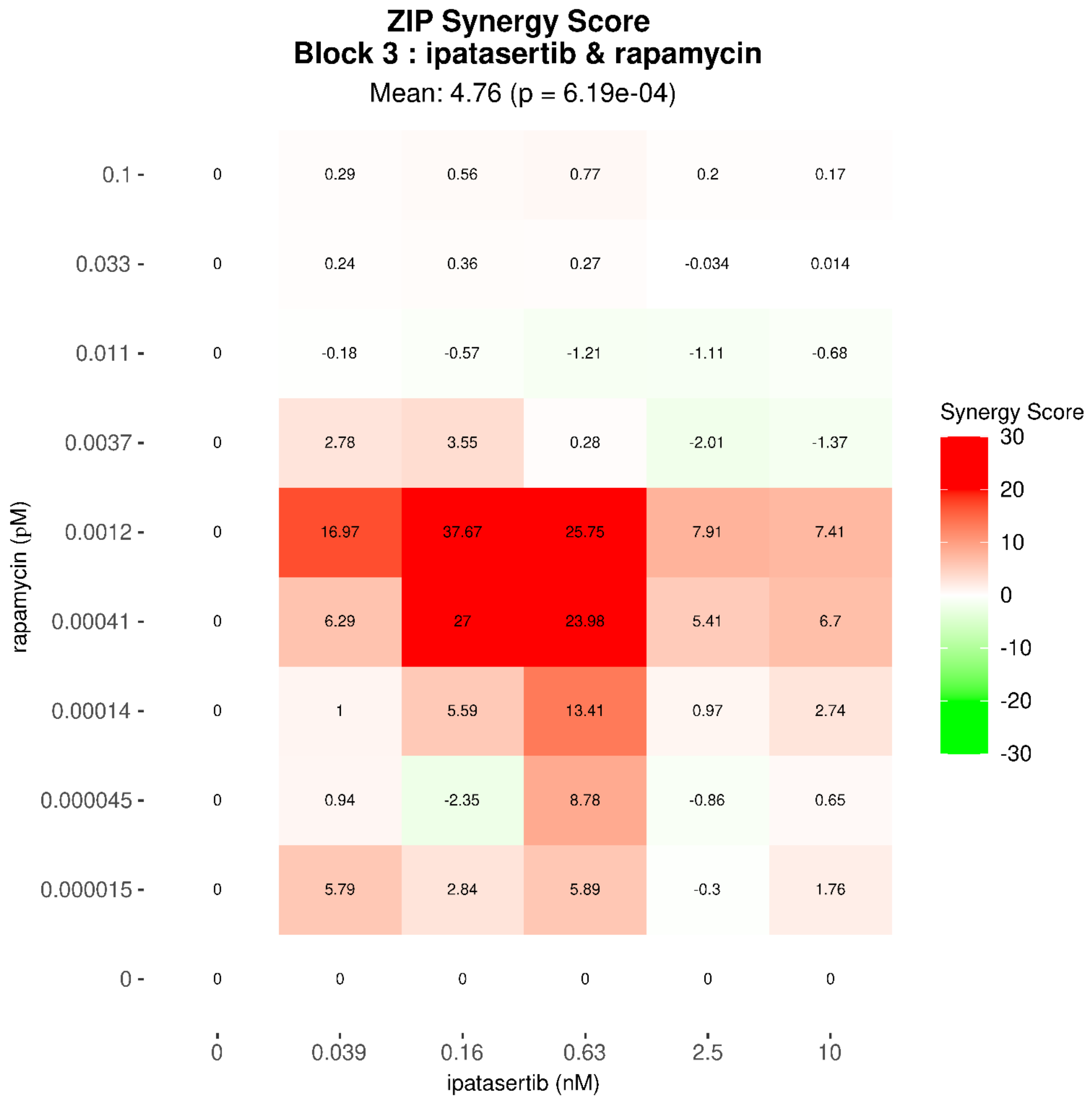

Table S18. Synergy scores for pairwise combination of ipatasertib and rapamycin in SUDHL-4 cells displayed in Fig. S6 (third replicate). Viable cell numbers were measured after 96 hours incubation. Synergy scores were calculation using zero interaction potency model (SynergyFinder3.0).

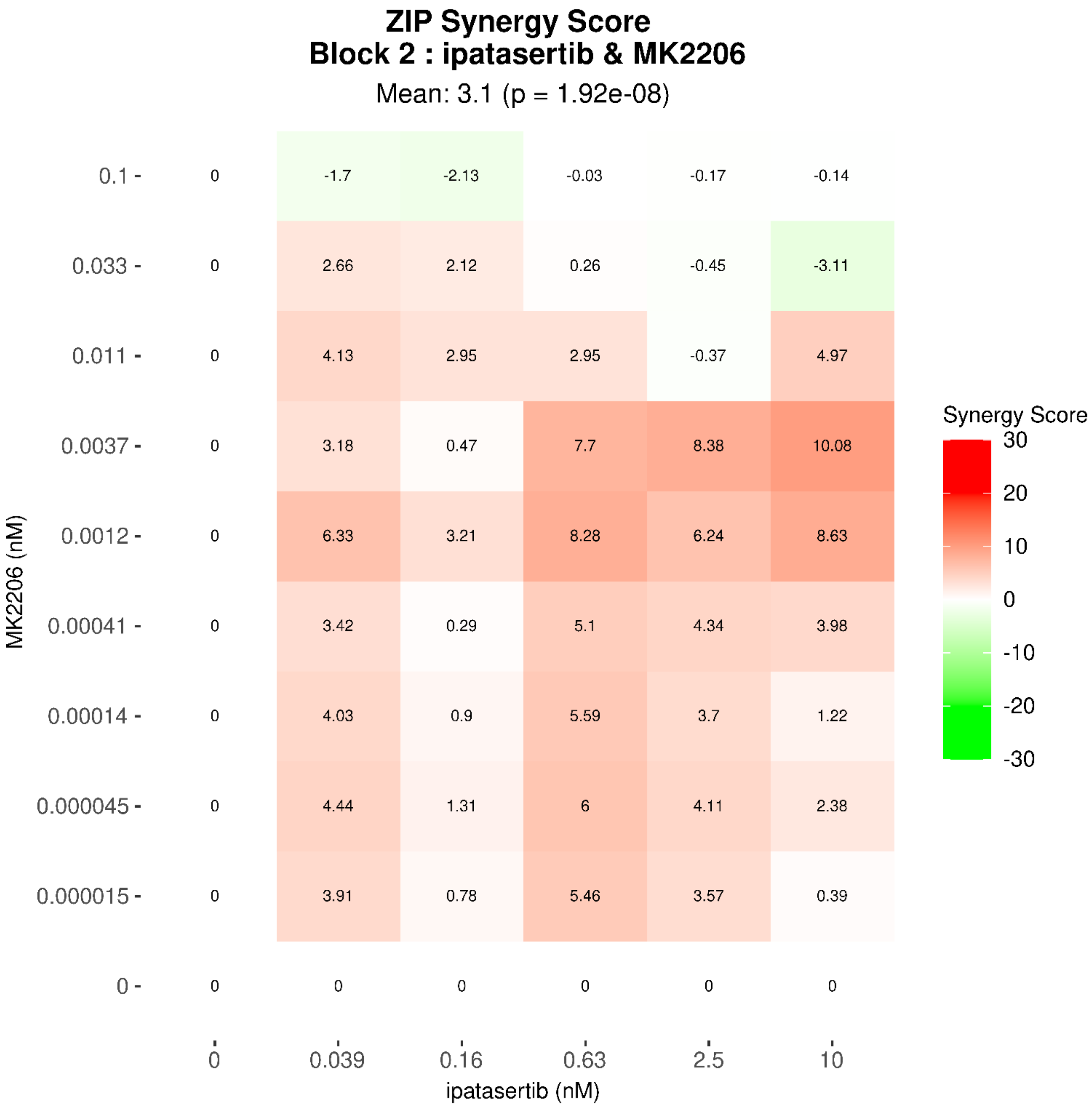

Table S19. Synergy scores for pairwise combination of MK2206 and ipatasertib in OCI-Ly7 cells displayed in Fig. S7. Viable cell numbers were measured after 96 hours incubation. Synergy scores were calculation using zero interaction potency model (SynergyFinder3.0).

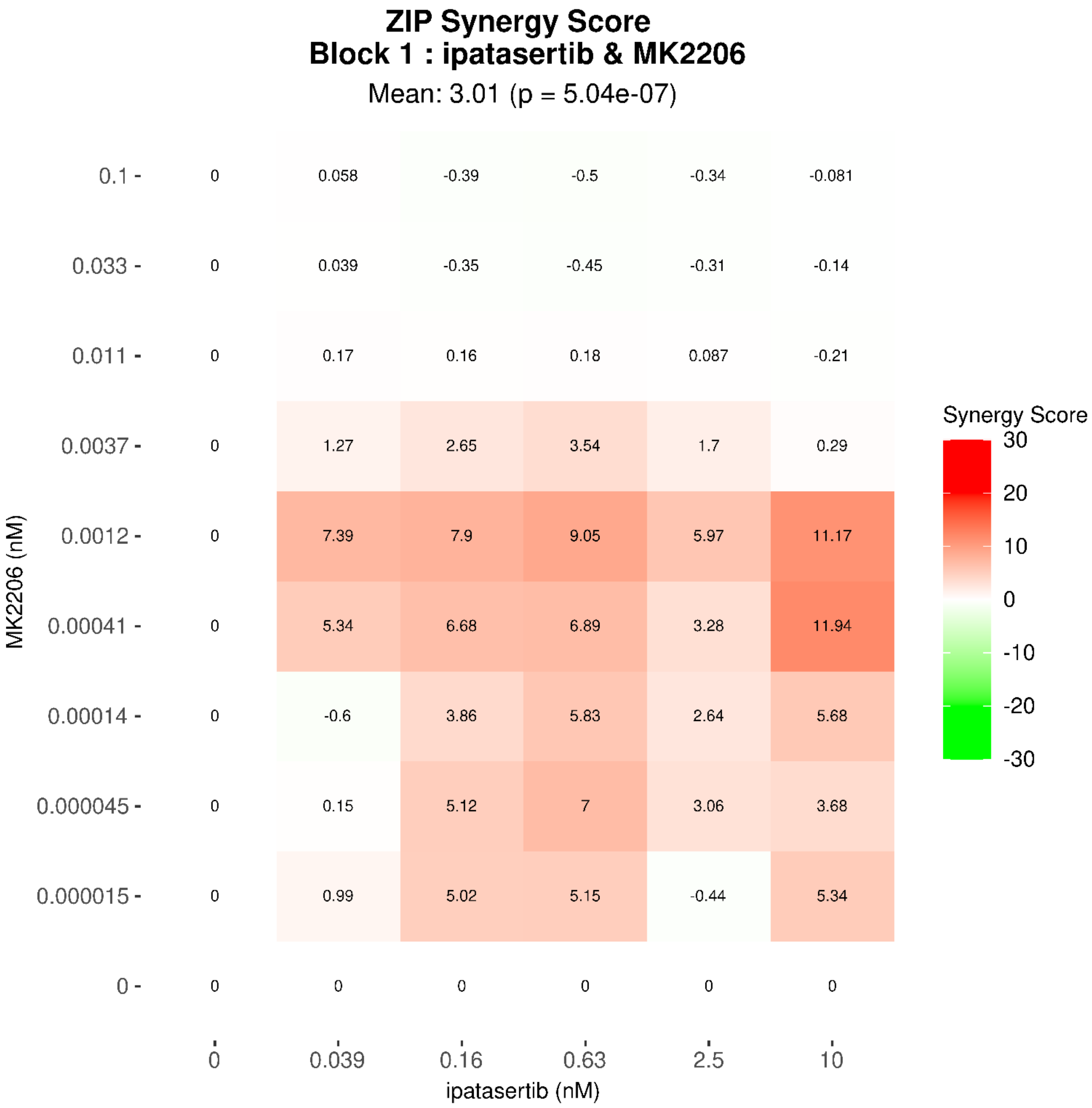

Table S20. Synergy scores for pairwise combination of MK2206 and ipatasertib in SUDHL-4 cells displayed in Fig. S7. Viable cell numbers were measured after 96 hours incubation. Synergy scores were calculation using zero interaction potency model (SynergyFinder3.0).

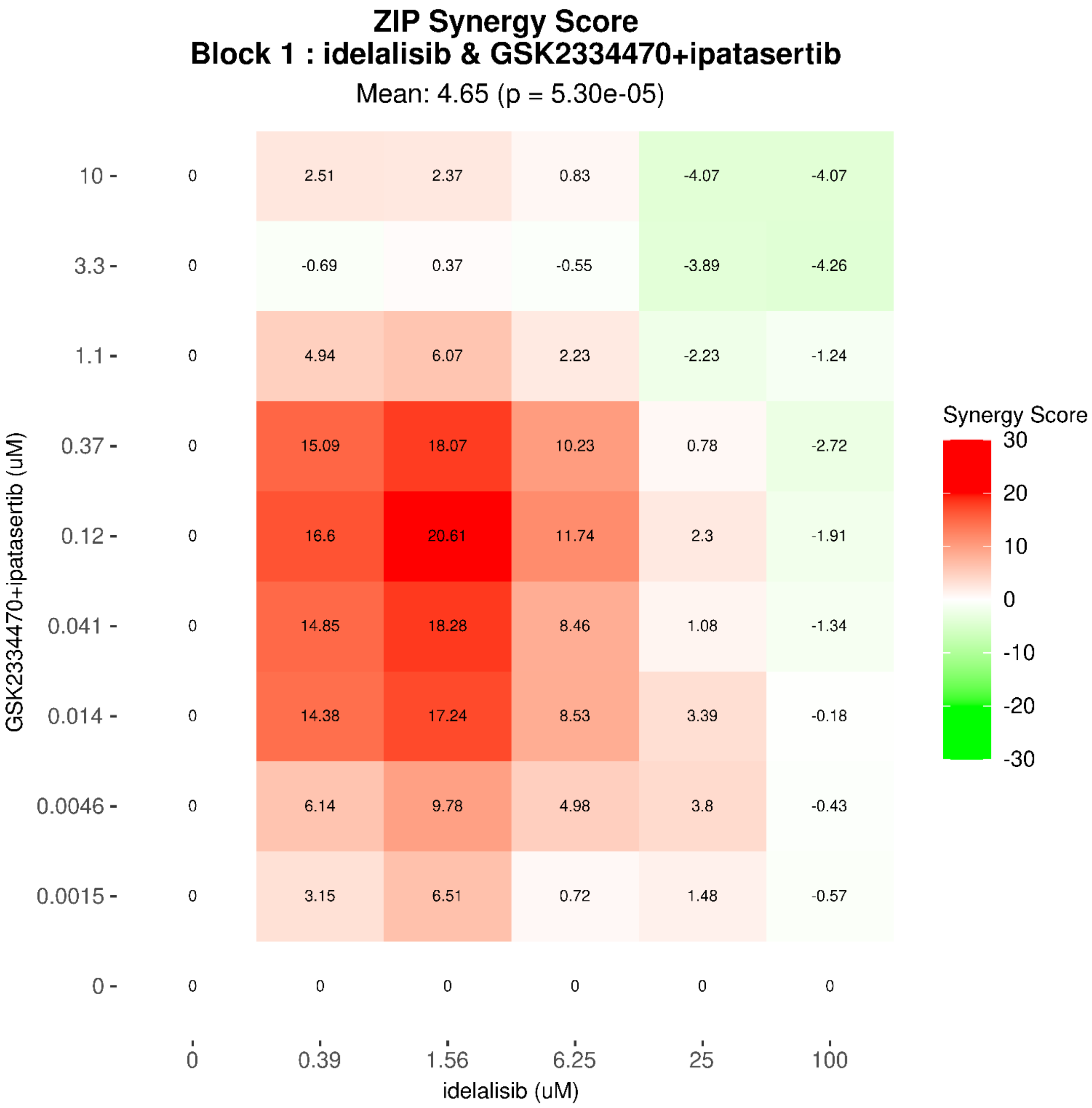

Table S21. Synergy scores for pairwise combination of GSK2334470/ipatasertib mix (in 2 : 1 molar ratio, respectively; concentrations are displayed for GSK2334470) and idelalisib in OCI-Ly7 cells displayed in Fig. S9. Viable cell numbers were measured after 96 hours incubation. Synergy scores were calculation using zero interaction potency model (SynergyFinder3.0).

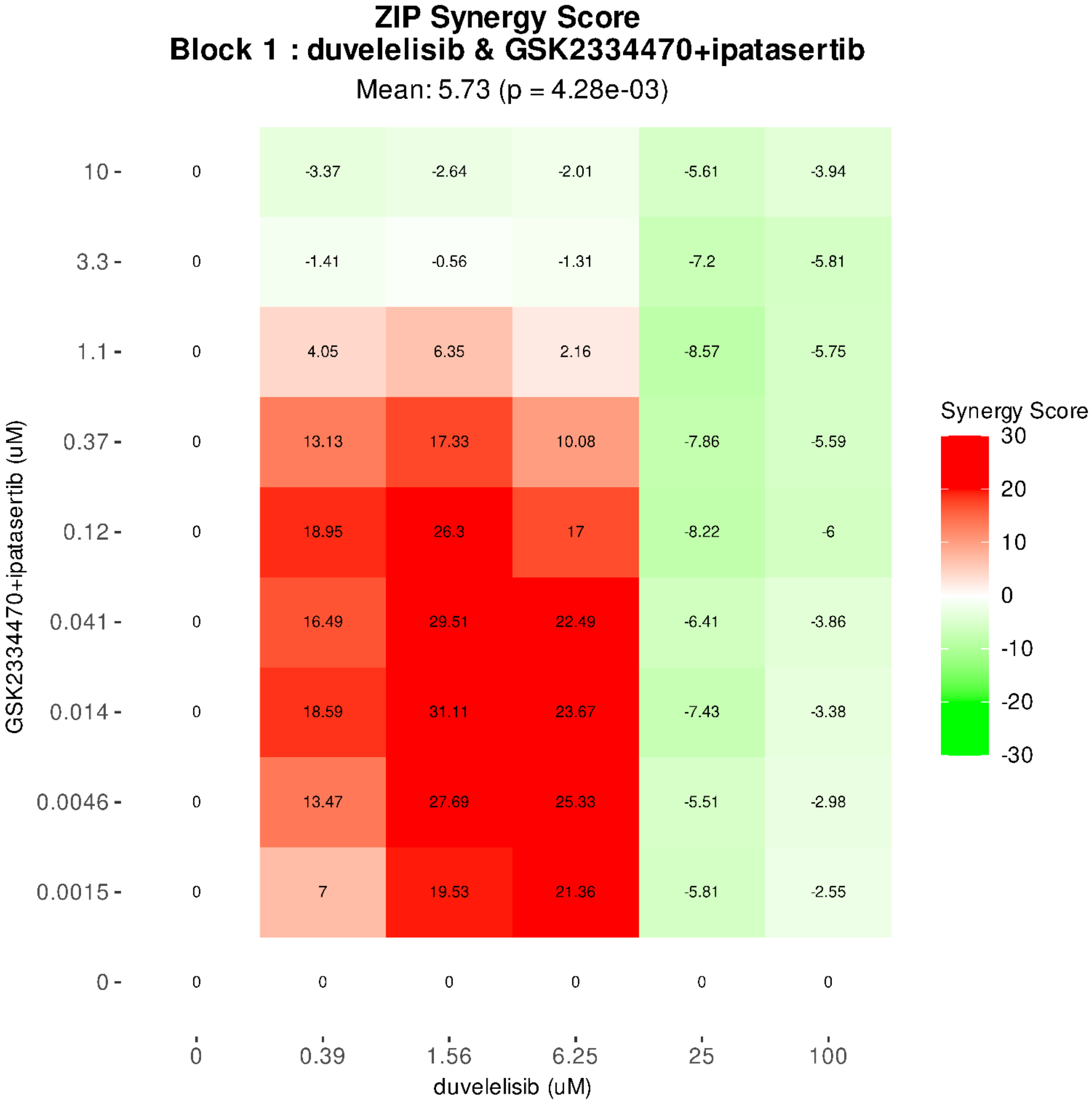

Table S22. Synergy scores for pairwise combination of GSK2334470/ipatasertib mix (in 2 : 1 molar ratio, respectively; concentrations are displayed for GSK2334470) and duvelisib in OCI-Ly7 cells displayed in Fig. S9. Viable cell numbers were measured after 96 hours incubation. Synergy scores were calculation using zero interaction potency model (SynergyFinder3.0).

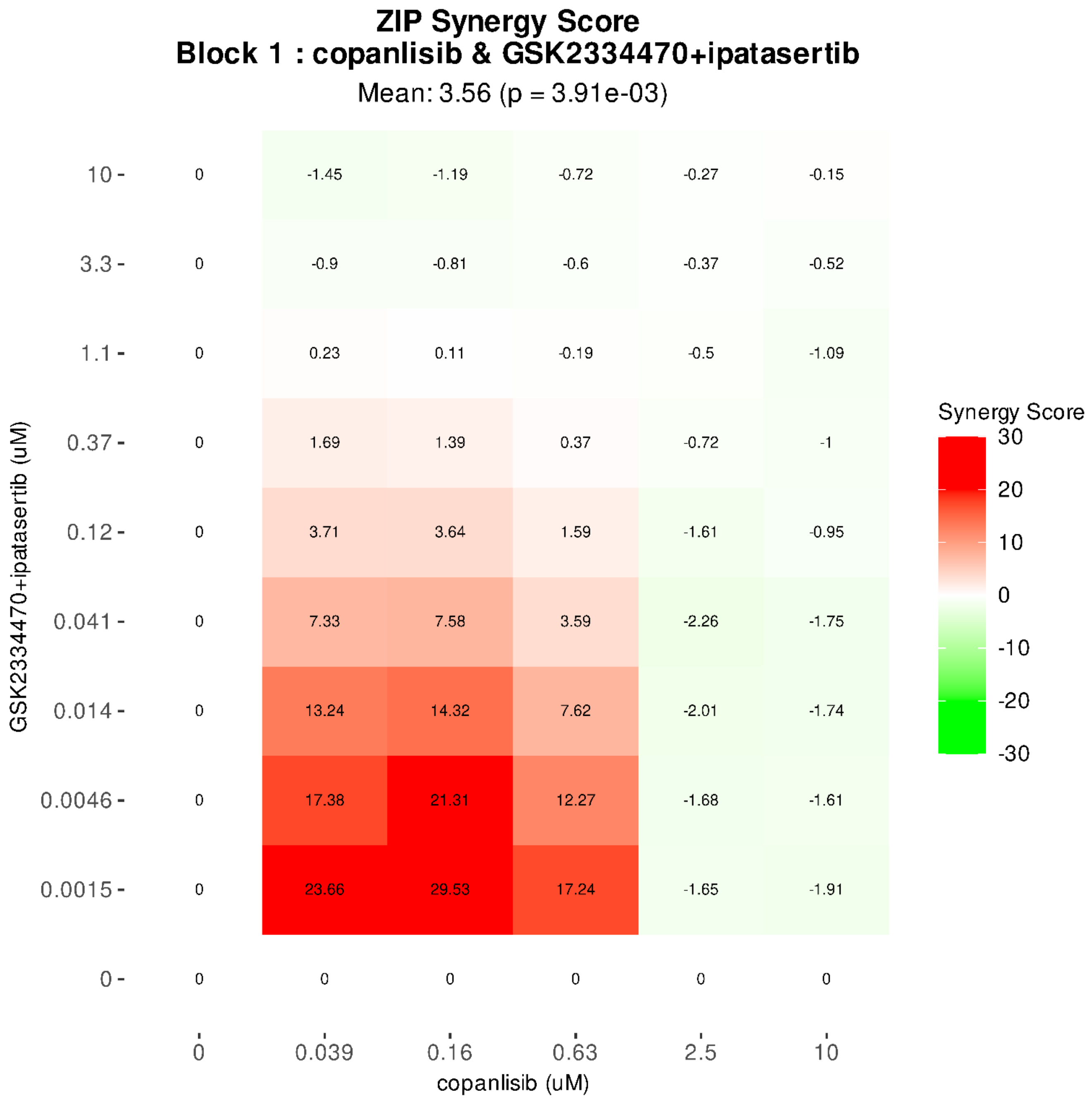

Table S23. Synergy scores for pairwise combination of GSK2334470/ipatasertib mix (in 2 : 1 molar ratio, respectively; concentrations are displayed for GSK2334470) and copanlisib in OCI-Ly7 cells displayed in Fig. S9. Viable cell numbers were measured after 96 hours incubation. Synergy scores were calculation using zero interaction potency model (SynergyFinder3.0).

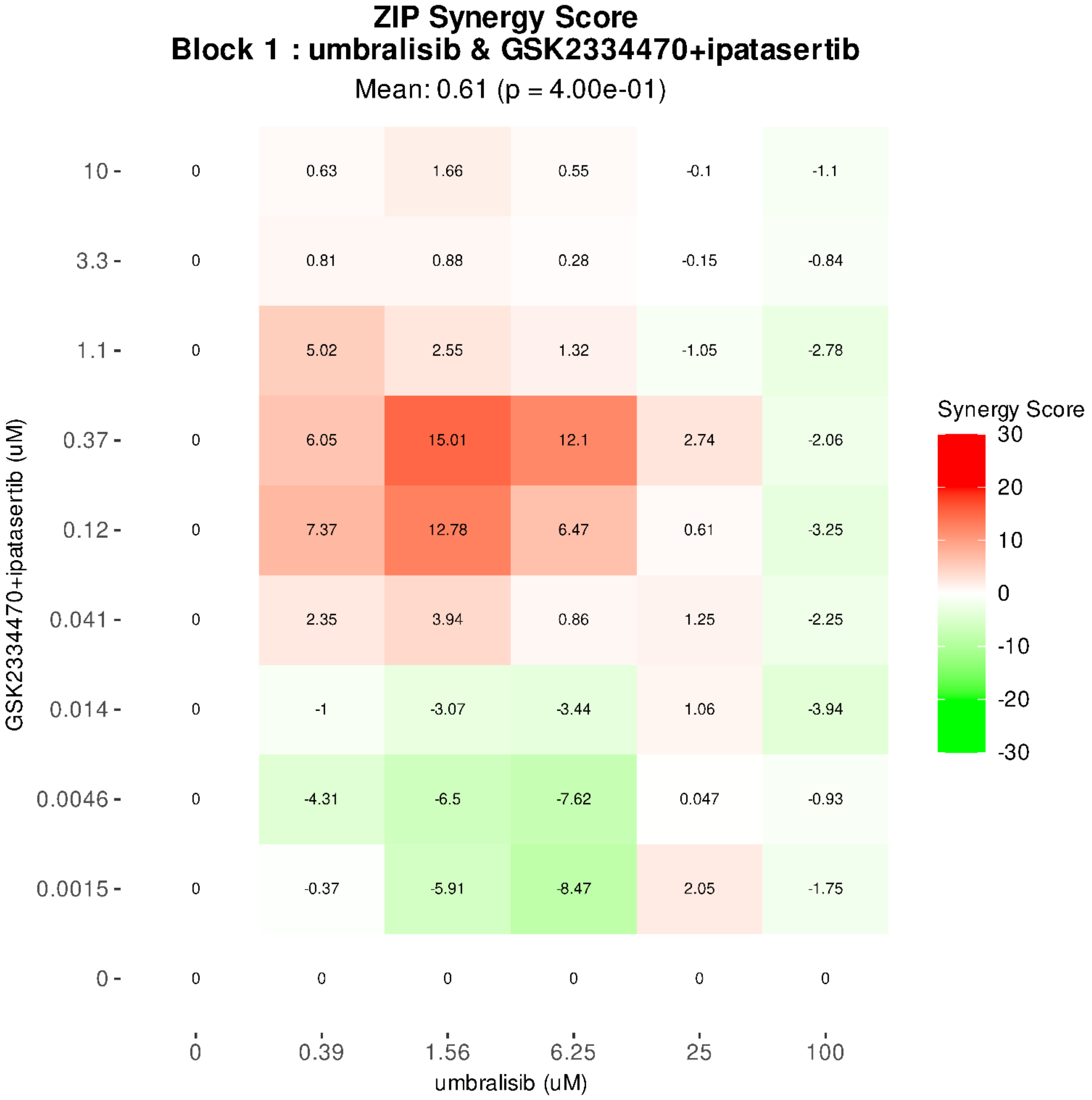

Table S24. Synergy scores for pairwise combination of GSK2334470/ipatasertib mix (in 2 : 1 molar ratio, respectively; concentrations are displayed for GSK2334470) and umbralisib in OCI-Ly7 cells displayed in Fig. S9. Viable cell numbers were measured after 96 hours incubation. Synergy scores were calculation using zero interaction potency model (SynergyFinder3.0).

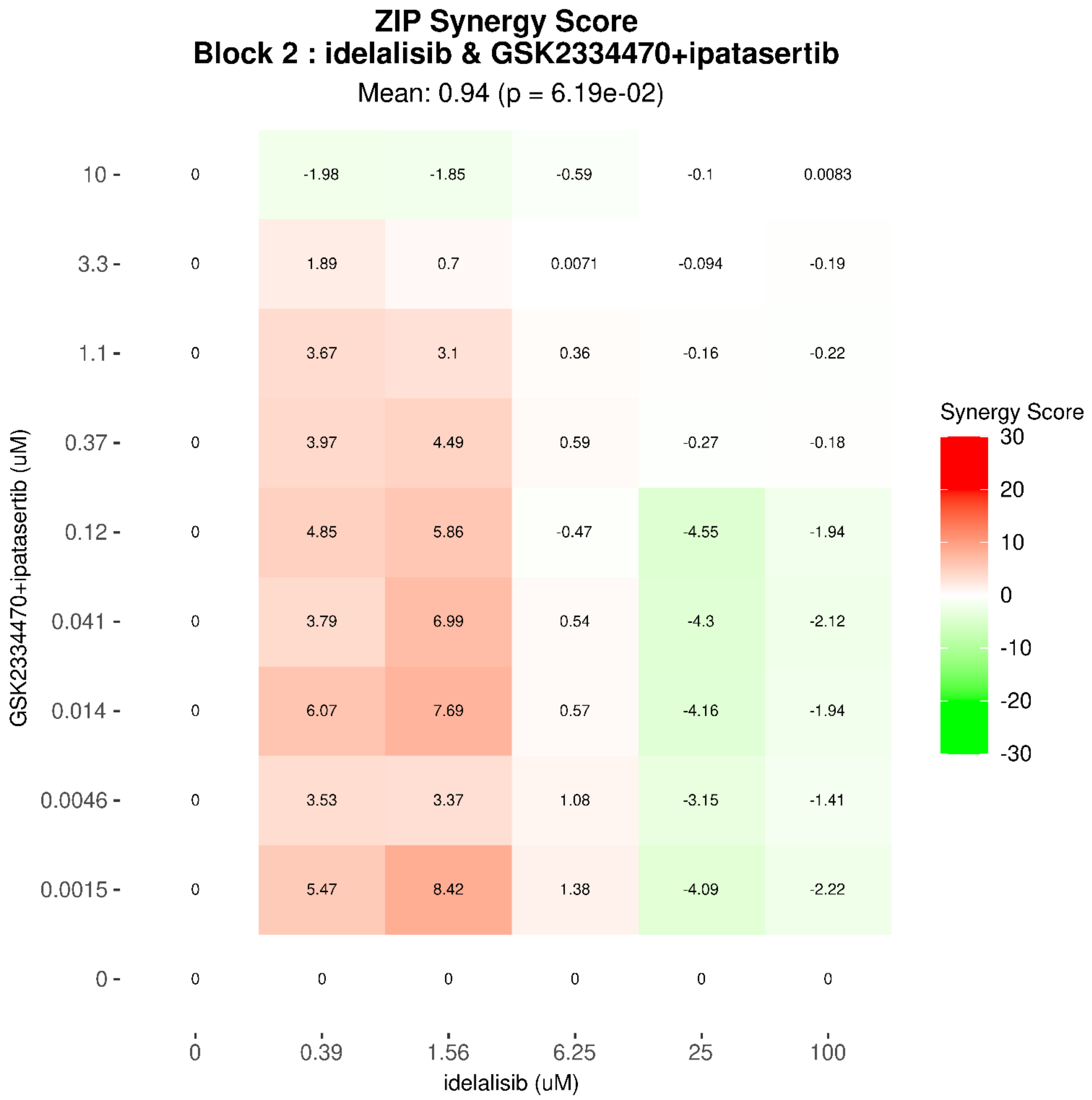

Table S25. Synergy scores for pairwise combination of GSK2334470/ipatasertib mix (in 2 : 1 molar ratio, respectively; concentrations are displayed for GSK2334470) and idelalisib in SUDHL-4 cells displayed in Fig. S9. Viable cell numbers were measured after 96 hours incubation. Synergy scores were calculation using zero interaction potency model (SynergyFinder3.0).

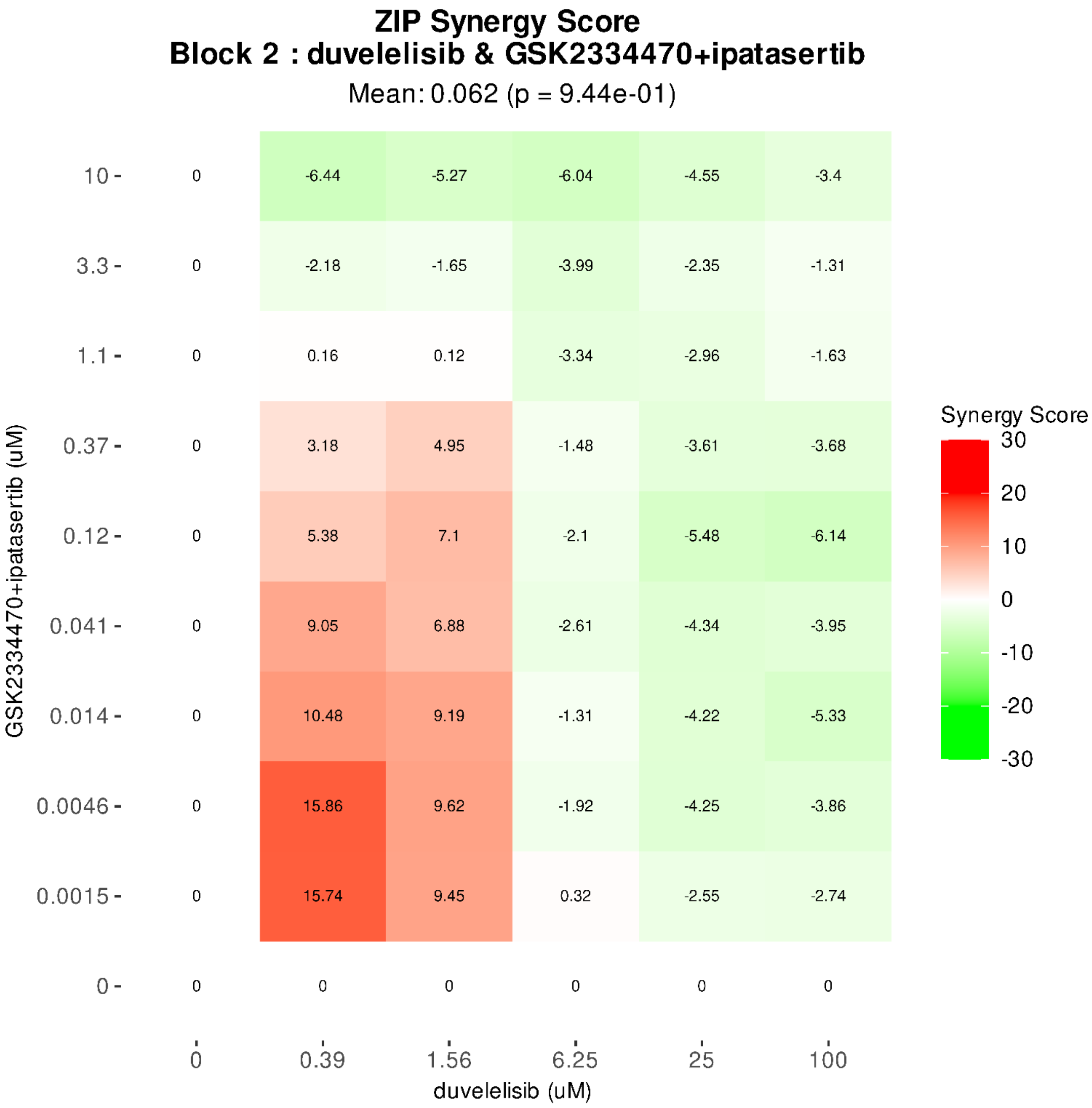

Table S26. Synergy scores for pairwise combination of GSK2334470/ipatasertib mix (in 2 : 1 molar ratio, respectively; concentrations are displayed for GSK2334470) and duvelisib in SUDHL-4 cells displayed in Fig. S9. Viable cell numbers were measured after 96 hours incubation. Synergy scores were calculation using zero interaction potency model (SynergyFinder3.0).

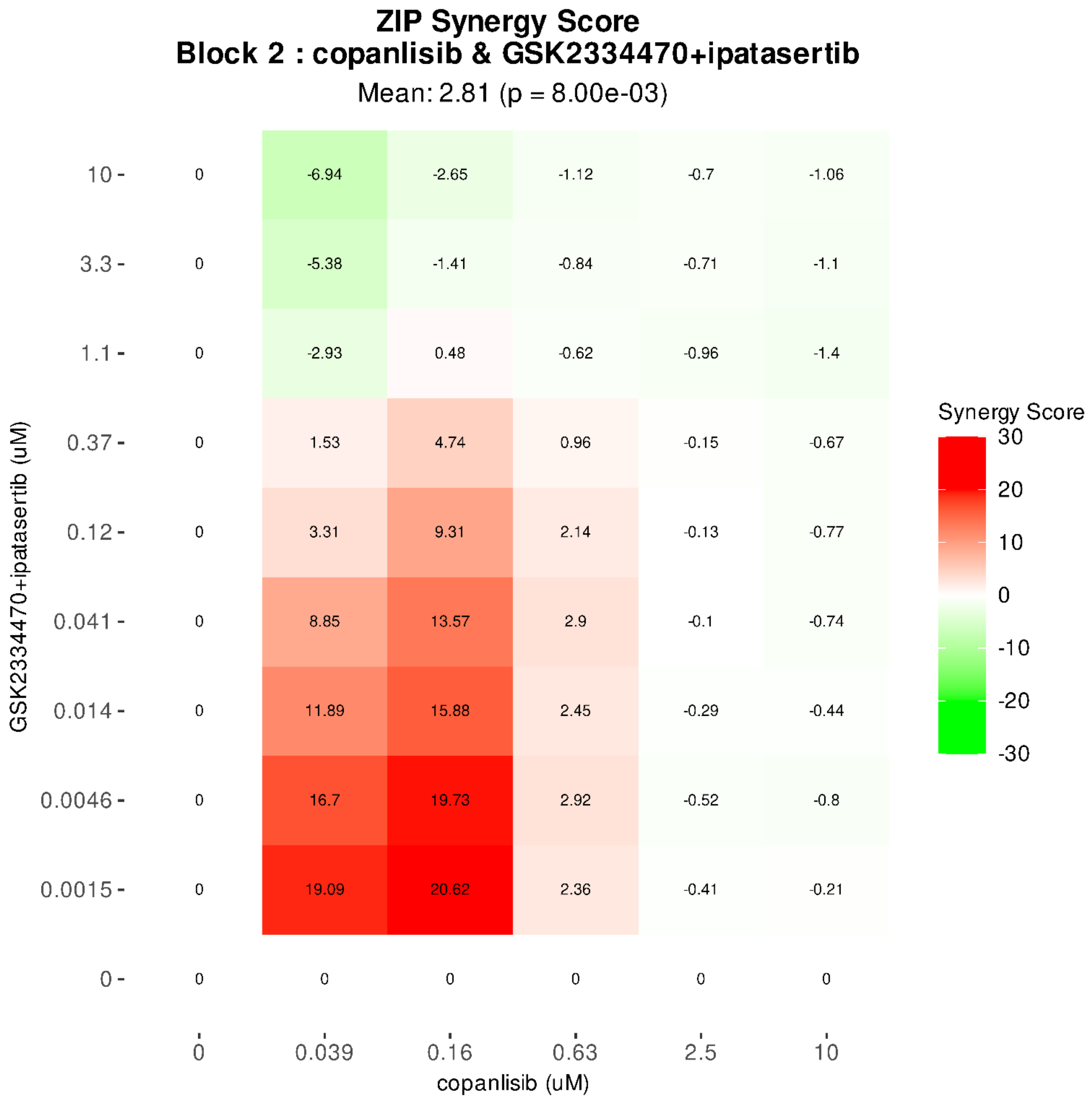

Table S27. Synergy scores for pairwise combination of GSK2334470/ipatasertib mix (in 2 : 1 molar ratio, respectively; concentrations are displayed for GSK2334470) and copanlisib in SUDHL-4 cells displayed in Fig. S9. Viable cell numbers were measured after 96 hours incubation. Synergy scores were calculation using zero interaction potency model (SynergyFinder3.0).

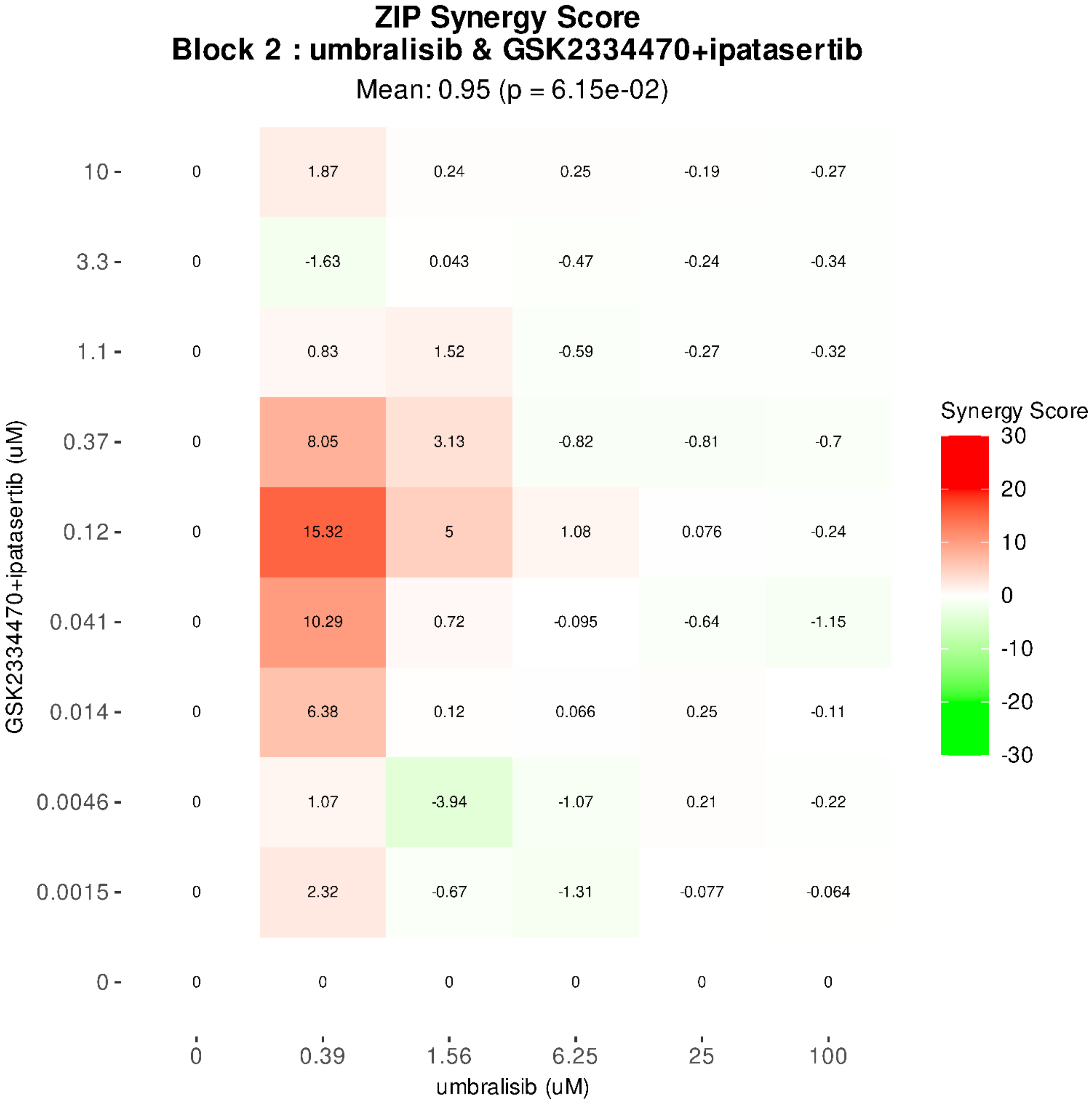

Table S28. Synergy scores for pairwise combination of GSK2334470/ipatasertib mix (in 2 : 1 molar ratio, respectively; concentrations are displayed for GSK2334470) and umbralisib in SUDHL-4 cells displayed in Fig. S9. Viable cell numbers were measured after 96 hours incubation. Synergy scores were calculation using zero interaction potency model (SynergyFinder3.0).
